# Stationary and portable multipollutant monitors for high-spatiotemporal-resolution air quality studies including online calibration

**DOI:** 10.5194/amt-14-995-2021

**Published:** 2021-02-09

**Authors:** Colby Buehler, Fulizi Xiong, Misti Levy Zamora, Kate M. Skog, Joseph Kohrman-Glaser, Stefan Colton, Michael McNamara, Kevin Ryan, Carrie Redlich, Matthew Bartos, Brandon Wong, Branko Kerkez, Kirsten Koehler, Drew R. Gentner

**Affiliations:** 1 Department of Chemical & Environmental Engineering, Yale University, School of Engineering and Applied Science, New Haven, Connecticut 06511, USA; 2 SEARCH (Solutions for Energy, Air, Climate and Health) Center, Yale University, New Haven, Connecticut 06511, USA; 3 Department of Environmental Health and Engineering, Johns Hopkins Bloomberg School of Public Health, Baltimore, Maryland 21205, USA; 4 Department of Mechanical Engineering, Yale University, School of Engineering and Applied Science, New Haven, Connecticut 06511, USA; 5 Department of Electrical Engineering, Yale University, School of Engineering and Applied Science, New Haven, Connecticut 06511, USA; 6 Department of Internal Medicine, Yale University, School of Medicine, New Haven, Connecticut 06510, USA; 7 Department of Environmental Health Sciences, Yale University, School of Public Health, New Haven, Connecticut 06511, USA; 8 Department of Civil, Architectural and Environmental Engineering, The University of Texas at Austin, Cockrell School of Engineering, Austin, Texas 78712, USA; 9 Civil and Environmental Engineering, University of Michigan, 2350 Hayward St, G.G. Brown Building, Ann Arbor, Michigan 48109, USA; 10 Multiphase Chemistry, Max Planck Institute for Chemistry, 55128 Mainz, Germany

## Abstract

The distribution and dynamics of atmospheric pollutants are spatiotemporally heterogeneous due to variability in emissions, transport, chemistry, and deposition. To understand these processes at high spatiotemporal resolution and their implications for air quality and personal exposure, we present custom, low-cost air quality monitors that measure concentrations of contaminants relevant to human health and climate, including gases (e.g., O_3_, NO, NO_2_, CO, CO_2_, CH_4_, and SO_2_) and size-resolved (0.3–10 μm) particulate matter. The devices transmit sensor data and location via cellular communications and are capable of providing concentration data down to second-level temporal resolution. We produce two models: one designed for stationary (or mobile platform) operation and a wearable, portable model for directly measuring personal exposure in the breathing zone. To address persistent problems with sensor drift and environmental sensitivities (e.g., relative humidity and temperature), we present the first online calibration system designed specifically for low-cost air quality sensors to calibrate zero and span concentrations at hourly to weekly intervals. Monitors are tested and validated in a number of environments across multiple outdoor and indoor sites in New Haven, CT; Baltimore, MD; and New York City. The evaluated pollutants (O_3_, NO_2_, NO, CO, CO_2_, and PM_2.5_) performed well against reference instrumentation (e.g., *r* = 0.66–0.98) in urban field evaluations with fast *e*-folding response times (≤1 min), making them suitable for both large-scale network deployments and smaller-scale targeted experiments at a wide range of temporal resolutions. We also provide a discussion of best practices on monitor design, construction, systematic testing, and deployment.

## 1 Introduction

Exposures to air pollution are associated with elevated health risks such as cardiorespiratory inflammatory responses and oxidative stress (Brauer et al., 2012; Chuang et al., 2007; Pope and Dockery, 2006). Each year outdoor air pollution leads to approximately 4.2 million premature deaths worldwide and is the fifth highest mortality risk factor in the Global Burden of Disease Study 2015 (Cohen et al., 2017; Forouzanfar et al., 2016). Assessment of public health risks and regulatory standards requires accurate measurement of air pollution levels. However, traditional analytical techniques for air pollutant measurements – such as spectroscopy, chemiluminescence, and mass spectrometry – are expensive, which limits the deployment of instruments to sparsely located state and federal air quality monitoring sites and targeted research campaigns. As a result, the spatiotemporal variations in urban human exposure caused by localized combustion sources (e.g., motor vehicles, cooking) and other sources are not well understood (Kheirbek et al., 2013).

The need for better geospatial coverage in air quality monitoring has resulted in multiple studies that utilize low-cost sensors to measure a range of pollutants in portable and stationary configurations (e.g., Bigi et al., 2018; Castell et al., 2017; Cross et al., 2017; Hagan et al., 2018; Kim et al., 2018; Lewis et al., 2016; Mead et al., 2013; Popoola et al., 2016; Thorson et al., 2019; Zimmerman et al., 2018). Wearable devices containing sensors to measure real-time gas-phase air pollutants such as nitric oxide (NO), nitrogen dioxide (NO_2_), carbon monoxide (CO), carbon dioxide (CO_2_), and ozone (O_3_) have been developed, but proper calibration still poses a challenge (Cao and Thompson, 2016; Mead et al., 2013). For example, Piedrahita et al. (2014) developed wearable air quality monitors (M-Pods) from primarily metal oxide semiconductor sensors. They demonstrated an ability to quantify ambient concentrations of CO, NO_2_, CO_2_, and O_3_ and found that field calibrations, given a wider range of environmental conditions, performed better than laboratory calibrations.

On a larger scale, environmental compliance and policymaking require an understanding of the air pollutant sources and their transport processes, and long-term high-density stationary monitoring networks are needed to fulfill this purpose. In 2010, Mead et al. (2013) deployed 46 sensor nodes in the Cambridge (UK) area for 2.5 months to measure NO, NO_2_, and CO. This study demonstrated the feasibility of using low-cost sensors to obtain environmental data at high spatiotemporal resolution. A more recent deployment of two Aerodyne ARISense systems collocated with state air quality stations was described by Cross et al. (2017). The study reported mixed performance of Alphasense electrochemical NO, CO, NO_2_, and O_*x*_ (*r*^2^ = 0.88, 0.84, 0.69, and 0.39) sensors at a 5 min temporal resolution. Numerous state, federal, and international programs continue to evaluate emerging sensor technologies, e.g., South Coast Air Quality Sensor Performance Evaluation Center (AQ-SPEC), EPA Air Sensor Toolbox, and the World Meteorological Organization (WMO). For example, the WMO provides a review of low-cost sensor first principles and descriptions of sensor drift and cross-sensitivities by measurement type, including information on many of the sensors used in this study (Lewis et al., 2018). While low-cost sensors have great potential to provide air quality data at higher spatiotemporal resolution and complement existing monitoring sites, multiple studies have reported measurement biases caused by sensor drift due to environmental variables and aging (Borrego et al., 2016; Cross et al., 2017; Lewis et al., 2016; Mead et al., 2013; Zimmerman et al., 2018). Hence, careful sensor characterization, calibration, and data processing are important to ensure measurement accuracy.

In this study we design and evaluate custom stationary and portable multipollutant monitors as part of the SEARCH (Solutions for Energy, AiR, Climate and Health) Center at Yale–Johns Hopkins, which will deploy the multipollutant monitors in a long-term, citywide network in Baltimore, MD, while also enabling applications in other studies (e.g., Schilling et al., 2020). Specifically we (a) describe the physical hardware design, sensors employed, and relevant testing with a focus on overcoming historical limitations; (b) describe and demonstrate the first online calibration system for low-cost monitors; (c) present laboratory tests and field measurements with our monitors to demonstrate real-world performance; (d) compare the performance of the multipollutant monitor to other low-cost monitor deployments; and (e) provide best practices for monitor design to enable future research.

## 2 Materials and methods

### 2.1 Instrument design

#### 2.1.1 Incorporated sensors

A suite of sensors is built into the SEARCH multipollutant stationary monitors to measure the concentration of CO, NO_2_, NO, CO_2_, O_3_, methane (CH_4_), size-resolved particulate matter (PM), and, when applicable, sulfur dioxide (SO_2_). It also collects relative humidity (RH) and temperature (*T*) data to correct for RH–*T* influences on sensor response during field deployment with changing environmental conditions. Here the term “monitor” or “multipollutant monitor” is used to describe the collection of sensors and other components (e.g., flow channels, valves, online calibration system) used while the term “sensor” is used to describe the standalone sensing components. Manufacturers and part numbers for the selected sensors are listed in Table 1. Due to size limitations, either a NO or SO_2_ sensor is included in the multipollutant monitor based on the application. The monitors evaluated in this study contain NO sensors to better characterize urban NO_*x*_ (NO and NO_2_) pollution. Monitors with SO_2_ sensors will be applied specifically in targeted emissions studies or future locations with higher SO_2_ concentrations. The CH_4_ and CO_2_ sensors are integrated to evaluate greenhouse gas emissions. The portable monitor integrates sensors for CO, CO_2_, NO_2_, O_3_, and PM to evaluate personal exposure in a space-efficient package.

Measurements of CO, NO_2_, NO, and SO_2_ are made using the four-electrode electrochemical parts-per-billion-level A4 series sensors from Alphasense (available at http://www.alphasense.com, last access: 4 January 2021). Different models of electrochemical sensors manufactured by Alphasense have been tested in previous studies (e.g., Hagan et al., 2018; Mead et al., 2013; Zimmerman et al., 2018) and demonstrate promise for ambient measurements with careful calibration and system design. We used the updated A431 model to measure O_*x*_ with the portable monitor. The four-electrode configurations were chosen over the three-electrode sensors because the extra auxiliary electrode (AE), which has the same functionality as the working electrode (WE) but is not exposed to the analyte, provides a background electrode response. When coupled together with the WE, this reduces the influences of RH–*T* on sensor signals. Two common concerns for electrochemical and metal oxide sensors are sensor drift over time and cross sensitivity to other pollutants. Electrochemical sensor drift due to sensor aging causes the change of calibration curve intercepts, and many studies have shown drift needs to be corrected for deployments over the course of several months (Cross et al., 2017; Hagan et al., 2018). Changes to sensor output by pollutants other than the target analyte are considered cross sensitivities. Pang et al. (2018) evaluated pollutant cross sensitivities for the Alphasense (B series) O_3_, SO_2_, CO, NO, and NO_2_ electrochemical sensors and found that while changes in RH were ultimately more important, cross sensitivities at ambient concentrations could contribute to 1%–10% of the target pollutant response if uncorrected. For example, they found that the CO electrochemical sensor had a small positive increase in WE voltage with exposure to O_3_ and NO_2_ while exposure to CO_2_ and SO_2_ decreased WE voltage.

The Alphasense nondispersive infrared (NDIR) sensor measures CO_2_, having an estimated limit of detection (LOD) of 1 ppm (Hodgkinson et al., 2013). The NDIR sensor has a broadband light source, and two bandpass filters centered at 4.26 and 3.95 μm. The 4.26 μm filter coincides with the CO_2_ absorption band centered at 4.2 μm. The 3.95 μm light is not absorbed by CO_2_ and works as a reference to account for potential drift in light intensity caused by lamp aging and power supply change. The CO_2_ sensor has similar dimensions as the A4 electrochemical sensors.

CH_4_ measurements are made using the Figaro TGS2600 gas sensor. Field evaluations of this sensor were performed by Eugster and Kling (2012) in Alaska. More recently, van den Bossche et al. (2017) conducted a systematic laboratory evaluation of a similar Figaro model. Both groups reported measurement agreement between the sensor and a reference technique after correcting for RH and temperature interferences. The Figaro TGS2600 is also sensitive to analytes such as CO, hydrogen (H_2_), and volatile organic compounds (VOCs), such as ethanol and isobutane (manufacturer’s specification). The cross sensitivity from CO can be corrected in the multipollutant monitor by using the onboard CO sensor. We remove the VOC interference by adding a layer of activated charcoal-impregnated cloth on top of the sensor to filter VOCs. The implementation and performance of this setup is detailed in Sect. 2.1.3.

For the stationary multipollutant monitor, the MiCS-2614 sensor measures O_3_ due to its proven past performance, low cost, and small size (5 × 7 × 1.55 mm). This sensor was built into a portable ozone monitor by Cao and Thompson (2016) where they found it agreed with 2B Technologies’ ozone monitor in the range of 20 to 100 ppb, with over-measurement under 20 ppb and under-measurement above 100 ppb. Note, at the time of this publication, this sensor is not being manufactured.

PM is measured with a miniature PM sensor PMS A003 produced by Plantower (available at http://www.plantower.com, last access: 4 January 2021). The sensor has an internal laser and uses scattered light to count particles and differentiate particle size. The device reports mass concentrations in PM_1_, PM_2.5_, and PM_10_ with precision of 1 μg m^−3^, as well as particle number concentrations for particle sizes bins: 0.3, 0.5, 1, 2.5, 5, and 10 μm. Levy-Zamora et al. (2019) demonstrated the ability of the sensor to perform under laboratory and ambient settings. With environmental correction factors, the sensor had an overall accuracy of 93% and an overall precision error of 10%. In a Baltimore co-location study, Datta et al. (2020) utilize the SEARCH multipollutant monitor with Plantower PMS A003 sensors to develop a multiple linear regression calibration with a 1 d average root-mean-square error of 2 μg m^−3^. In addition to concerns about environmental factors, it has been shown that the chemical composition of aerosols influences the performance of the Plantower sensor (Levy-Zamora et al., 2019).

#### 2.1.2 Electrical system

The electronics for the multipollutant monitor are designed to have modularized functions on each individual circuit board. Each sensor has its designated analog circuitry to supply power, amplify signals, and filter noise. The analog signals are fed to analog-to-digital converters (ADCs) on the daughter boards to minimize noise pick-up in the wiring or other circuitry.

The Alphasense electrochemical sensors are powered with potentiostatic circuitries with zero bias for the CO, NO_2_, and SO_2_ sensors and a 200mV bias for the NO sensor. Special care was taken to match the input impedance for the NO potentiostatic circuit to minimize noise. The circuit amplification is designed to output an analog signal of approximately 1V per 100 ppb NO, SO_2_, and NO_2_ and 10 ppm CO. The on-board ADC sequentially converts the amplified and filtered signals generated by the AE and the WE. The AE voltage is recorded as the background signal, and the differential signal between WE and AE voltages is used as the sensor signal for calibration and measurement purposes.

The CO_2_ sensor is driven with a 2 Hz 5V 50% duty cycle waveform clocked by a MEMS (microelectromechanical systems) oscillator. The outputs of the CO_2_ sensor are two DC-biased sinusoidal waves from the reference and active channels, and subsequent circuitries are implemented to remove the DC offset and amplify the signals. Two peak detection circuits are applied to sample and hold the peak heights of the two amplified sinusoidal waves to be read sequentially by the ADC. This design uses significantly less processing resources, in comparison with continuous sampling and peak detection through software.

The CH_4_ and O_3_ circuitries are placed on one circuit board to conserve space and accommodate mechanical requirements (Sect. 2.1.3). These two sensors function by changing their resistances when exposed to their corresponding analytes. Hence, voltage dividers with low-temperature-coefficient load resistors were applied, and the sensor resistances can be derived by sampling the voltages across the load resistors through ADCs.

The humidity and temperature (RH–*T*) sensor is placed on a separate small circuit board and towards the front of the inlet to minimize the influence of heat generated by the voltage drop across circuit board traces in the presence of other components. The PM sensor is equipped with a circuit board to convert from its 1mm pitch connection to a more convenient 2.54mm pitch connection to facilitate assembly. The RH–*T* sensor and the PM sensor both output digital signals, and the signals are acquired by the microcontroller directly. Daughter boards for the portable multipollutant monitor are combined or miniaturized versions of the stationary design in order to reduce the amount of wiring and required space.

A central control board generates sensor input voltages; powers components on–off (such as solenoid valves to perform calibration and background measurement); powers a piezoelectric blower (to circulate ambient air for the gas sensors); and reads, processes, stores, and transmits sensor data. The control processes use the Cypress 68-pin PSoC 5LP microcontroller, which interfaces with sensors through digital communication peripherals (I2C and UART). The data acquisition frequencies are set as follows: the NO_2_, NO, SO_2_, and CO sensors are sampled every 160 ms, with AE and WE signals each taking up 80 ms sequentially; the CH_4_ and O_3_ sensors are sampled every 160 ms, utilizing only one signal channel; the RH–*T* sensor is sampled every 160 ms for either RH data or temperature data sequentially, making their actual sampling period 320 ms; the CO_2_ sensor is sampled with 2 Hz frequency in accordance with the input drive frequency for both the active and reference channels; and the PM sensor is sampled every 640ms, to accommodate its low data output rate relative to the other components. See Table S1 for more information regarding the electronic system.

#### 2.1.3 Mechanical design

**Figure 1.**
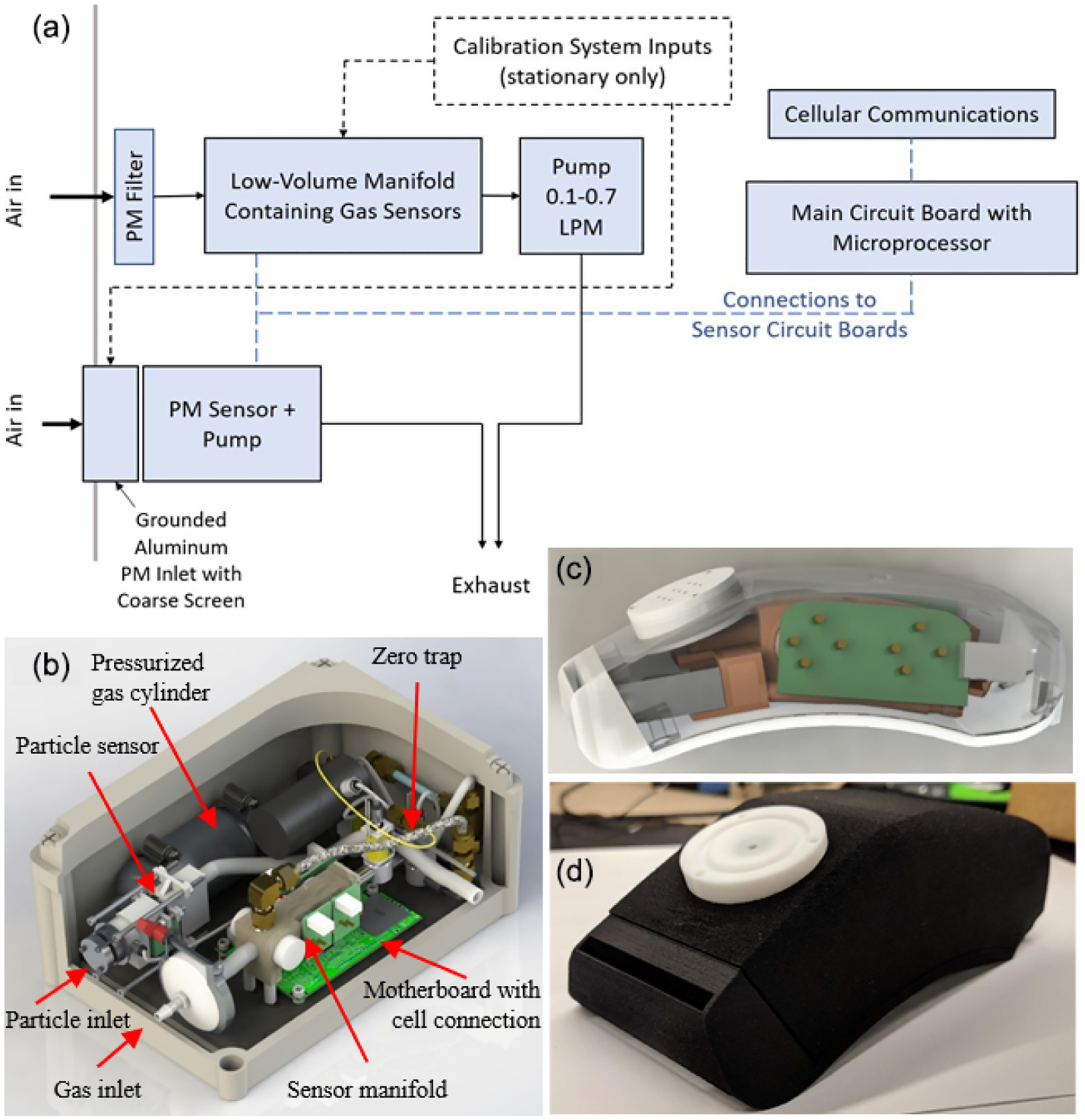
Monitor designs shown as (**a**) a simplified flow and electronics diagram, which is used in the (**b**) stationary (28×18×14 cm) and (**c**–**d**) portable (15×6.5×5 cm) versions of the multipollutant monitoring device. Panels (**b**–**c**) are SolidWorks renderings, and (**d**) is a photo. See Fig. S3 for additional photos.

The sampling manifold is designed to isolate the sensing areas of the gas sensors in a small active flow area separated from the rest of the device components (Figs. 1 and S3). The manifold is 3D-printed with WaterShed XC11222 resin through stereolithography (SLA), which prints materials with a dense, gastight finish. Other 3D printing materials such as acrylonitrile butadiene styrene (ABS) and polylactic acid (PLA) were also tested. These materials are often printed with the fused deposition modeling (FDM) method, creating porous parts that need surface treatment of acetone to be gastight. To minimize the potential shape deformation resulting from post-printing treatment, we use the SLA method to print the manifold and other 3D printed parts of the device. O-ring grooves are incorporated in the manifold to secure and provide an airtight seal for the sensors. To minimize potential ozone loss, the ozone sensor is placed closest to the manifold inlet. In our testing with a 2B-Tech reference ozone monitor, the ozone loss rate is 4%–12% for XC11122 resin, versus 7%–22% for ABS. To further reduce losses of reactive analyte, a PTFE liner is inserted into the inlet of the XC11122 manifold to reduce contact between the sampling air and the manifold material. The outlet of the manifold is connected to the piezoelectric blower that sampled at an average flow rate of 0.6 standard liters per minute (slpm). To optimize monitor response time, the pollutant exchange rate in the manifold is maximized with high flow rates and a small internal volume of ~9 mL, producing an estimated 1 s residence time in the manifold. The ambient air entering the manifold is first pulled through a filter holder with a 2 μm thick, 47 mm diameter Teflon filter to keep the inside of the manifold clean of particles and collect filter samples for offline analysis.

The CH_4_ sensor inside the manifold is covered by a layer of activated carbon-impregnated cloth (Zorflex^®^Double Weave), which is secured by a 3D-printed PLA cylindrical shell. It is then wrapped in Teflon tape in order reduce pollutant interactions with PLA inside of the manifold. This activated carbon cloth layer is effective in filtering out VOC interference for the sensor. For instance, when covered by the activated carbon cloth, the CH_4_ sensor did not respond to ethanol concentrations as high as 2%. Even after continuous exposure to outdoor VOC for 3 months, with the activated carbon cloth cover, the CH_4_ sensor did not respond to ethanol vapor when an open vial was placed near it. For comparison, when the CH_4_ sensor without the used activated carbon cloth was placed directly above an open vial of ethanol, sensor resistance dropped by approximately 5 kΩ equivalent to 0.3 ppm methane. While such highly concentrated ethanol vapors are less common in the ambient environment, the activated carbon filter is also likely effective for other VOCs at lower concentrations. Here, ethanol is specifically tested as the challenge compound because the sensor is known to be highly responsive to ethanol, and activated carbon is a known, effective hydrocarbon filter for a wide range of VOCs. All calibrations used for in-field concentrations include the use of the filter.

Inlet and outlet enclosures are designed for the PM sensor to direct air flow (Fig. 1). Specifically, the inlet enclosure contained an SLA 3D-printed Watershed XC11122 holder to support the sensor and an aluminum inlet, through which sample air flows into the sensor inlet. Aluminum was chosen over 3D-printed plastic material as the inlet duct and grounded to the motherboard to avoid electrostatic particle losses due to static charges on a non-metallic surface. The front of the aluminum duct is covered with an aluminum disk placed 30 mm above it, between which a 32×32 mesh stainless-steel wire cloth is installed to block insects and large dust particles. Ambient air flows through the screen and enters the aluminum channel to reach the sensor inlet. The aluminum disk is placed above the inlet to block light, which was shown to interfere with normal operation and cause the sensor to output PM mass concentrations above 3000 μg m^−3^. To reduce the intrusion of light and water, the device is installed with both the gas and particle inlets pointed downwards.

The portable monitor makes all measurements immediately adjacent to the breathing zone with all sensors contained in a small custom shoulder-mountable housing that is 3-D printed and easily attachable to a bag, backpack, purse, or other strap (Fig. 1c and d). A small auxiliary enclosure (23 × 12 × 6.5 cm; 1 kg including battery) is required to house the re-chargeable battery and main circuit board. The design of the gas sensor manifold is similar to that of the stationary monitor with a piezoelectric blower promoting fast air exchange rates in a minimal volume manifold with PM removed at the inlet via a 23 mm PFTE filter in a PTFE housing. PM is measured via a separate minimal inlet with a light shield.

#### 2.1.4 Online calibration and zero system

All monitors undergo multi-point calibrations under variable, realistic RH–*T* conditions in an environmental chamber prior to field installation. To improve data quality during field deployment and to better track and correct for sensor drift, the stationary monitor includes a laboratory-tested span calibration and zero system. These systems are not incorporated into the portable model to conserve space and minimize weight. However, portable monitors will be periodically calibrated in the environmental chamber over the course of the SEARCH project. These features are aimed at helping with long-term deployment of the multipollutant monitors. Due to the shorter deployment schedule for this study, we focus only on the calibration and zero system efficacy in laboratory and selected field deployments for proof of concept.

The calibration process has either two or three calibration functions depending on the configuration, with the ability to change the temporal frequency at which each occurs. Each stationary monitor has zeroing functions for both the PM sensor and the gas sensor suite while half of the monitors for the SEARCH deployment also have a gas span calibration function using a miniaturized standard cylinder (dependent upon cylinder composition). In the SEARCH project, the PM and gas zeroing functions are scheduled to occur twice a week and the gas span calibration once a week. Depending on the inclusion of the standard gas cylinder, two or three three-way solenoid valves are placed in the system to direct flows and alternate normal ambient sampling with PM zero, gas zero, and gas span calibration functions (flow diagram shown in Fig. S4).

For the PM zero, the exhaust from the piezoelectric blower of the gas system, in which particles have been filtered out by a Teflon filter, is directed to the aluminum inlet of the PM sensor. This results in an overflow to the PM inlet due to the higher flow rate of the gas system relative to the particle system, producing a mass concentration of zero.

Zero concentrations for the gas sensors are obtained using either a filtering “zero trap” or via their absence from the calibration cylinder, which is primarily nitrogen and specifically useful for compounds that do not have room-temperature filtration options available for the zero trap. A series of scrubbing materials are used to remove select gas-phase analytes: soda lime for CO_2_ and activated carbon and stainless-steel wool for O_3_. To obtain the zero-concentration signals with the zero trap, the exhaust of the piezoelectric blower is passed through the packed materials directed to the gas sensors through a side port on the manifold near the inlet. The flow rate through the packed tube is 50 standard cubic centimeters per minute (sccm). With the 9mL internal volume, the air inside the manifold is re-circulated and passed through the packed tube 16 times in 3 min to ensure complete analyte removal. At the time of writing, we were unable to find materials to effectively remove CH_4_, CO, NO_2_, and NO at ambient temperatures. Therefore, their zero-concentration signals are determined in the laboratory with zero air prior to field deployment and are checked routinely with the balance of zero air in the calibration cylinder (with the exception of CO and CH_4_, which are calibrated via the CO present in the cylinder).

The standard gas delivery system is designed to overflow the manifold with known concentrations of gas standard from a miniature stainless-steel gas cylinder (2 in. o.d.×5.5 in. height, Swagelok) that is filled with 5 ppm CO and 2000 ppm CO_2_ to 1500 psig in a balance of nitrogen from a primary authentic cylinder (Airgas). This approach produces a single calibration span value similar to that produced during lab calibration. Prior to installation, the pressure regulator (Tescom) is adjusted, in combination with a 0.006–0.007 in. i.d. PEEK constriction (1*/*16 in. o.d.), to deliver > 30 sccm of standard gas flow into the manifold through the exhaust port of the piezoelectric blower with the blower off. Delivery at or above that flow rate is used to ensure constant overflow conditions regardless of cylinder pressure. The components used for the gas delivery system were selected to withstand the pressures of the cylinder and environmental temperatures encountered in the enclosure. Due to concerns over long-term chemical stability of certain reactive gases (e.g., NO), only CO and CO_2_ are included in the cylinder. The concentrations of the standard gases in the cylinder are also designed to avoid acute exposure concerns. Future work must consider these safety aspects and, given the use of high-pressure gases, should consult with environmental health and safety representatives about any related aspects.

A water permeation setup is included in the standard gas delivery line to maintain a minimum humidity inside the manifold during calibration and prevent unrealistically dry conditions that skew electrochemical sensor response. The water permeation device includes a 3*/*8 in. diameter PTFE membrane (25.4 μm thick, McMaster-Carr) installed in a stainless-steel tee (Swagelok) where the membrane separates the standard gas flow from a reservoir of deionized water. The PTFE membrane restricts direct water flow but allows for sufficient permeation of H_2_O molecules to raise the humidity of the dry standard gas to a level (>40% in this study) that allows for acceptable calibration conditions. Furthermore, with field operation, RH conditions during calibration can be routinely monitored using the RH–*T* sensor reporting via the data network. By varying the time of day during which the online calibration occurs, a wide range of realistic RH–*T* values can be used for assessing sensor drift and to adjust calibration curves.

#### 2.1.5 Cellular communications and data storage

All raw sensor data are written to a local SD card at subsecond frequency. Every 10 s the data are averaged and transmitted to a database hosted on a cloud server through an onboard 4G Telit LE910C1-NS cellular module. To achieve fast and continuous sensor data collection while maintaining simultaneous cellular data transmission, a task preemptive scheduler implemented within the microcontroller firmware tracks the status of the sensors and cellular module and executes core processes (e.g., read, write, send, and receive) at pre-set time intervals. This ensures that all sensor measurements are prioritized over other operating system tasks.

The data streams stored on the SD card and the cloud server include differential, working, and/or auxiliary channels for electrochemical and NDIR sensors, resistances for metal oxide sensors, size-resolved PM mass and number concentration, power supply voltage, and diagnostic information for calibration processes. The InfluxDB time series database (available at https://www.influxdata.com/, last access: 4 January 2021) is used to store, receive, and serve sensor data from field-deployed monitors. To maximize data availability, the database is hosted on an Amazon Web Services Elastic Compute Cloud (AWS EC2) instance (available at https://aws.amazon.com/, last access: 4 January 2021). The open-source data visualization platform Grafana (available at https://grafana.com/, last access: 4 January 2021) allows users and stakeholders to see real-time field-deployed monitor data remotely through a web browser (see Fig. S5 for screenshots of these data platforms).

### 2.2 Instrument evaluation

Our multipollutant monitor undergoes two phases of evaluation in this study: laboratory chamber experiments and ambient co-location with reference instruments. The laboratory experiments provide an isolated environment for characterizing sensor performance and establishing signal-response-to-concentration calibration curves. Outdoor ambient co-location experiments with reference instrumentation test the performance of the monitors over extended periods of time under a variety of real environmental conditions.

For laboratory calibration experiments at Johns Hopkins University, multipollutant monitors are placed inside a custom-built steel chamber (0.71 × 1.35 × 0.89 m). Environmental conditions range from 5%–85% humidity (most occurring around 30%–50%) and 20–40°C. Each gas pollutant is introduced into the chamber through filtered air inlets and diluted to a variety of concentrations above and below typical urban ambient levels using zero air (see Table 2). PM_1_, PM_2.5_, and PM_10_ are evaluated using the methodology presented in Levy-Zamora et al. (2019). Online calibration system tests and other sensor response tests at Yale University involve supplying authentic gas standards (Airgas) to the multipollutant monitor inlet or a similar sensor housing. See the SI for linear calibration data (Figs. S6 and S7) examples and information on how concentration values are calculated for electrochemical sensors.

Field evaluation took place at three different locations, dependent on the availability of reference instruments for inter-comparison. They included a site near an arterial roadway on Yale’s campus in New Haven, CT (Wall St.; 26 March–7 April 2018); Baltimore, MD, at the State of Maryland Department of the Environment Oldtown site (Oldtown Fire Station, 1100 Hillen St.; 18 May–7 June 2017, November–December 2017, and 14 June–12 July 2018); and in New York, NY (23 June 2018), and Baltimore, MD (2 March 2019), for separate tests of the portable monitor.

The temporal resolution of the comparison between the multipollutant monitor and reference instrumentation is primarily limited by the reference instrument. For NO_2_, PM_2.5_, and CO_2_ evaluations made at the Oldtown site in MD, the lowest temporal resolution of the reference is 1 h. For the New Haven, CT, field evaluations we use an on-site 2B-Tech (model 202) for O_3_ and Thermo Scientific model 48i for CO and are able to show comparisons at finer time resolution: 1 min for O_3_ and 10 min for CO. For comparison to the other sensors and literature, O_3_ and CO are also reanalyzed at a 1 h average. In New Haven, 1 h NO reference data are used from the Connecticut Department of Energy and Environmental Protection (DEEP) Criscuolo Park site.

Common metrics for evaluating the performance of sensor configurations include linear regression parameters between RH–*T* -corrected sensor data and reference instrument data such as the coefficient of correlation (*r*), coefficient of determination (*r*^2^), slope (*m*), and intercept (*b*). Ideal sensor performance would show strong correlation (*r* = 1 or −1, *r*^2^ = 1) as well as minimal over- or underestimation of the true concentration (*m* = 1*, b* = 0). Statistical error tests such as the mean bias error (MBE), mean absolute error (MAE), and root-mean-square error (RMSE) are also commonly used. The MBE represents the tendency for the sensor to over- or underestimate the reference, although positive and negative errors can cancel each other out. To get around that limitation, the MAE is similar to the MBE but looks only at the average absolute difference between the sensors. Finally, the RMSE represents how narrow the error distribution is by penalizing large measurements errors. All tests are reported in concentration units, which allows for physical interpretation of sensor performance. They are calculated as follows:

(1) $$\text{MBE}=\frac{1}{n}\sum_{i=1}^{n}\left(C_{i}^{\text{MPM}}-C_{i}^{\text{ref}}\right),$$

(2) $$\text{MAE}=\frac{1}{n}\sum_{i=1}^{n}\left|C_{i}^{\text{MPM}}-C_{i}^{\text{ref}}\right|,$$

(3) $$\text{RMSE}=\sqrt{\frac{1}{n}\sum_{i=1}^{n}{\left(C_{i}^{\text{MPM}}-C_{i}^{\text{ref}}\right)}^{2}},$$

where *n* is the total number of co-location data points, *C*^MPM^ is the concentration value of the multipollutant monitor, and *C*^ref^ is the concentration value of the reference monitor.

## 3 Results and discussion

### 3.1 Stationary monitor field results

#### 3.1.1 Particulate matter (PM_2.5_)

**Figure 2.**
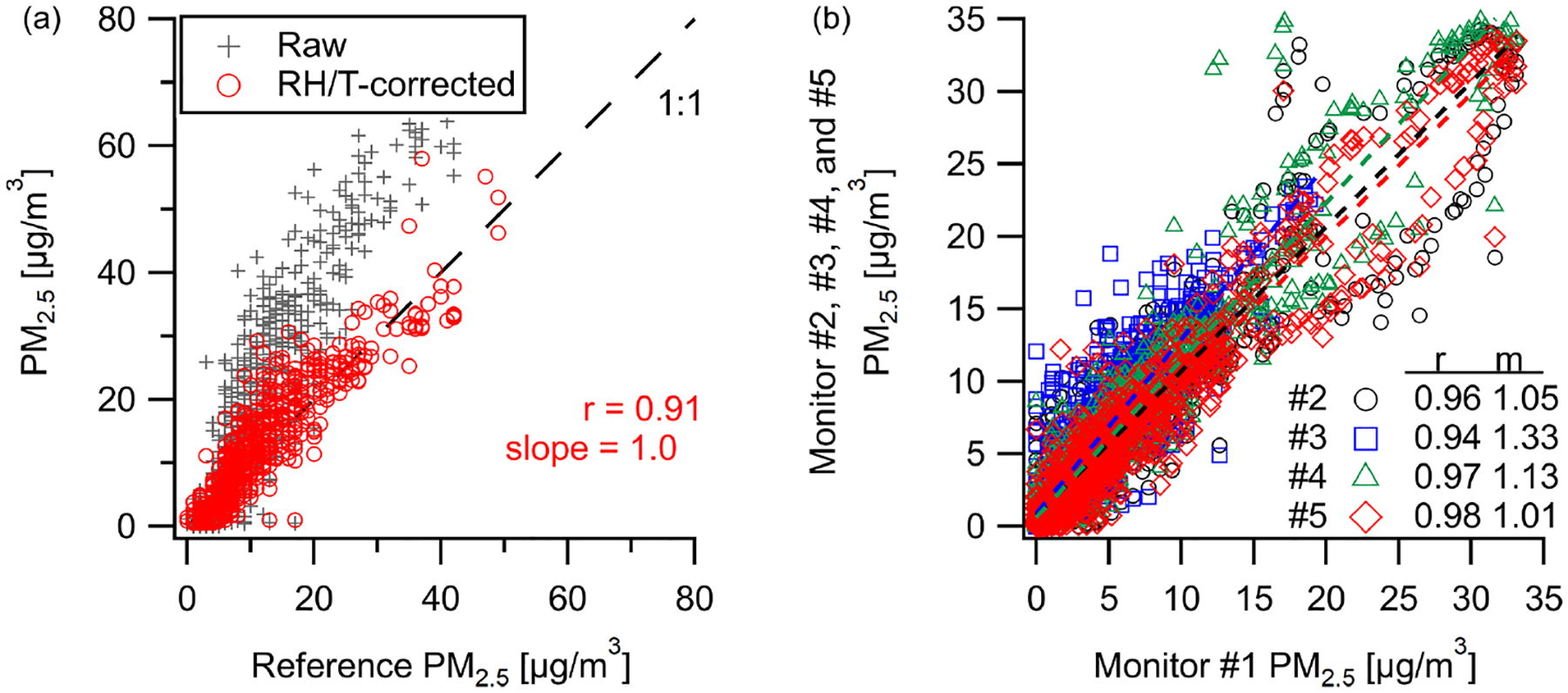
Comparison of PM_2.5_ concentrations between (**a**) our monitor and the Baltimore Oldtown site reference measurements and (**b**) an intercomparison of five co-located PM sensors over 2.5 weeks in New Haven where there is a high degree of correlation with measurements even at 10 min resolution (time series data can be found in Fig. S8).

Comparison of the Plantower PM sensor at the Oldtown site (Baltimore, MD) shows strong correlation (*r* = 0.91*, m* = 1.0) for PM_2.5_ with a 1 h averaging window over the span of 4 weeks. Figure 2a shows a clear overestimate in raw PM_2.5_ by the multipollutant monitor at higher concentrations, also as reported in Levy-Zamora et al. (2019) for the Plantower sensor, where we derive a RH–*T* correction equation to reduce bias and error (in this study: MBE = +0.9 μgm^−3^, RMSE = 4.3 μgm^−3^). At a separate New Haven, CT, deployment, our PM_2.5_ measurements are well-correlated (*r* = 0.94–0.98, *m* = 1.01–1.33) between five different multipollutant monitors at a 10 min resolution (Fig. 2b). See Fig. S8 for the time series data from Fig. 2b.

#### 3.1.2 Nitrogen dioxide

**Figure 3.**
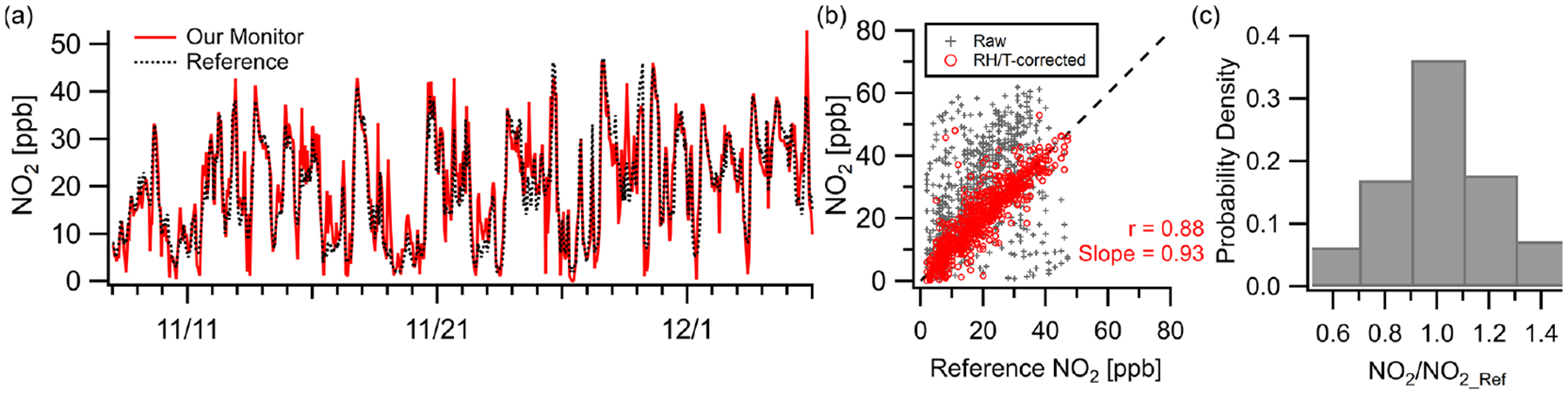
Outdoor ambient monitor comparison of NO_2_ from Baltimore, MD (Oldtown site). (**a**) Time series of RH–*T* -corrected multipollutant monitor data with reference data. (**b**) With RH–*T* correction factors the data achieve good correlation. (**c**) Over 35% of the measurements are within 10% of the reference site.

The NO_2_ sensor exhibited strong correlation (*r* = 0.88, *m* = 0.93) at the Oldtown site at hourly resolution. The monitor tracked well with the reference during both clean periods and periods of pollution maxima (MBE = +0.8 ppb, RMSE = 5.3 ppb), with concentrations ranging from near zero to over 50 ppb during the deployment (Fig. 3a). Correlation values between the raw multipollutant monitor data and the reference are significantly improved using a RH–*T* correction (Fig. 3b; see the Supplement for RH–*T* correction procedures). In addition, 35% of data points fall within 10% of the reference instrument, and 70% fall within 30% (Fig. 3c). The NO_2_ sensor is known to be cross sensitive to O_3_, but the NO_2_ sensors are manufacturer-equipped with an O_3_ filter rated to withstand 500 h at 2 ppm (or longer at lower concentrations). While the NO_2_ sensor did not exhibit cross sensitivity during the Oldtown deployment due to low ozone concentrations, future deployments should take the rating of the ozone filter into consideration and routinely monitor for biases due to ozone.

#### 3.1.3 Carbon monoxide

**Figure 4.**
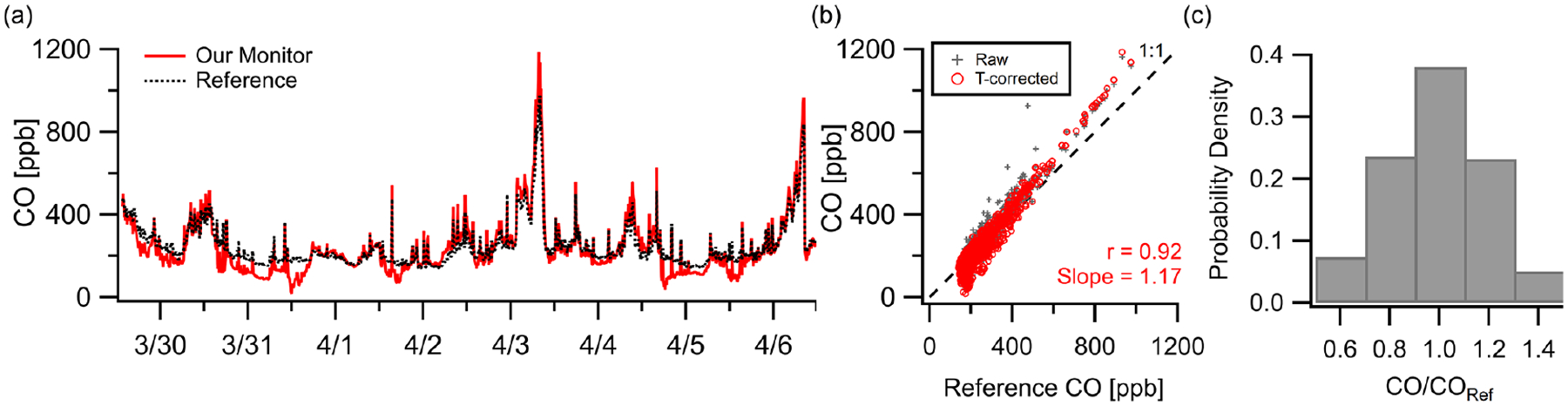
(**a**) Outdoor ambient monitor comparison of temperature-corrected CO data from New Haven, CT, with reference data. (**b**) Minimal overall effects from RH–*T* were observed (although the effect of *T* could be amplified at higher ambient levels; see Fig. S9). (**c**) At a 10 min resolution 38% of data points were within 10% of the reference and 85% within 30%.

The CO sensor demonstrated strong correlation (*r* = 0.92, *m* = 1.2) in a week-long deployment in New Haven, CT, at 10 min resolution. Figure 4a shows good tracking of pollution events and background shifts where concentrations exceeded 400 ppb but a noticeable underreporting relative to the reference during these relatively clean periods (< 200 ppb). These deviations coincided with elevated temperatures inside the monitor (> 15 °C) which is consistent with the zero temperature dependence listed by the manufacturer (Alphasense CO-A4, 2019a). To correct some of this offset, two sets of temperature corrections were used: a linear fit for all readings above 18 °C and a linear fit for all readings below 18 °C. Logarithmic and quadratic fits were tested for the high temperature relationship, but the best fit was linear. See Fig. S9 for the low- and high-temperature data points. Figure 4b shows that even with the correction factors, the overall trend and error (MBE = +5 ppb, RMSE = 59 ppb) remain similar to the raw data. After these corrections 43% of data points were within 10% of the reference, and 88% were within 30% at a 10 min resolution (Fig. 4c).

#### 3.1.4 Carbon dioxide

**Figure 5.**
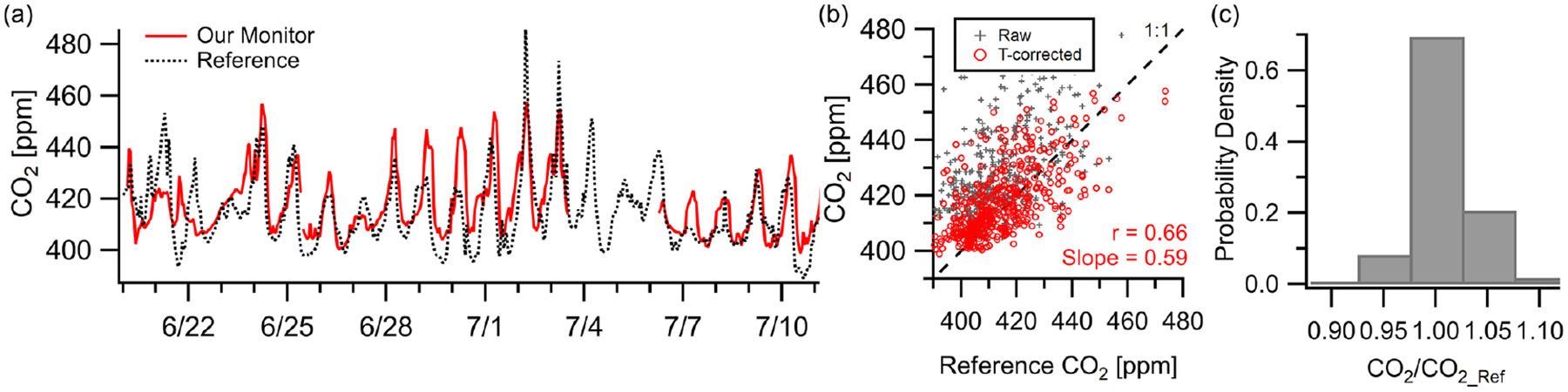
(**a**–**b**) Outdoor ambient monitor comparison of temperature-corrected CO_2_ data from Baltimore, MD, with reference data. The monitor was located at the OWLETS campaign while the reference data came from the NIST NEB site (2.7 km away). Occasional lags in pollution episodes, potentially due to the displacement of the monitor from the reference, are seen (e.g., 6/30, 7/1, 7/7), leading to a lower correlation coefficient than other pollutants. (**c**) Despite this, 70% of data points fall within 2.5% of the reference.

CO_2_ showed moderate correlation (*r* = 0.66*, m* = 0.59) with the NIST Northeast Corridor Urban Test Bed project’s NEB tower site over a 3-week deployment at a 1 h resolution. While not a direct co-location (2.7 km apart), the NEB site is used as a reference to examine citywide CO_2_ levels and trends while acknowledging that spatial differences from local sources may limit the inter-comparison due to vertical or horizontal variance. Figure 5a shows the monitor trends well with the reference after temperature correction, although the monitor occasionally exceeds the reference concentration by 10–20 ppm (MBE = +3.4 ppm, RMSE = 11 ppm). This is consistent with the reported accuracy of ~15 ppm shown in the manufacturer data sheet at 400 ppm in laboratory testing (Alphasense IRC-A1, 2018). Possible lags in regional pollution episodes can be observed in the time series data (e.g., 6/30, 7/1, 7/7), leading to a lower correlation value than other presented pollutants. Despite this, 70% of readings were within 2.5% of the reference instrument (~10 ppm) and 98% were within 7.5% (~ 30 ppm).

#### 3.1.5 Ozone

**Figure 6.**
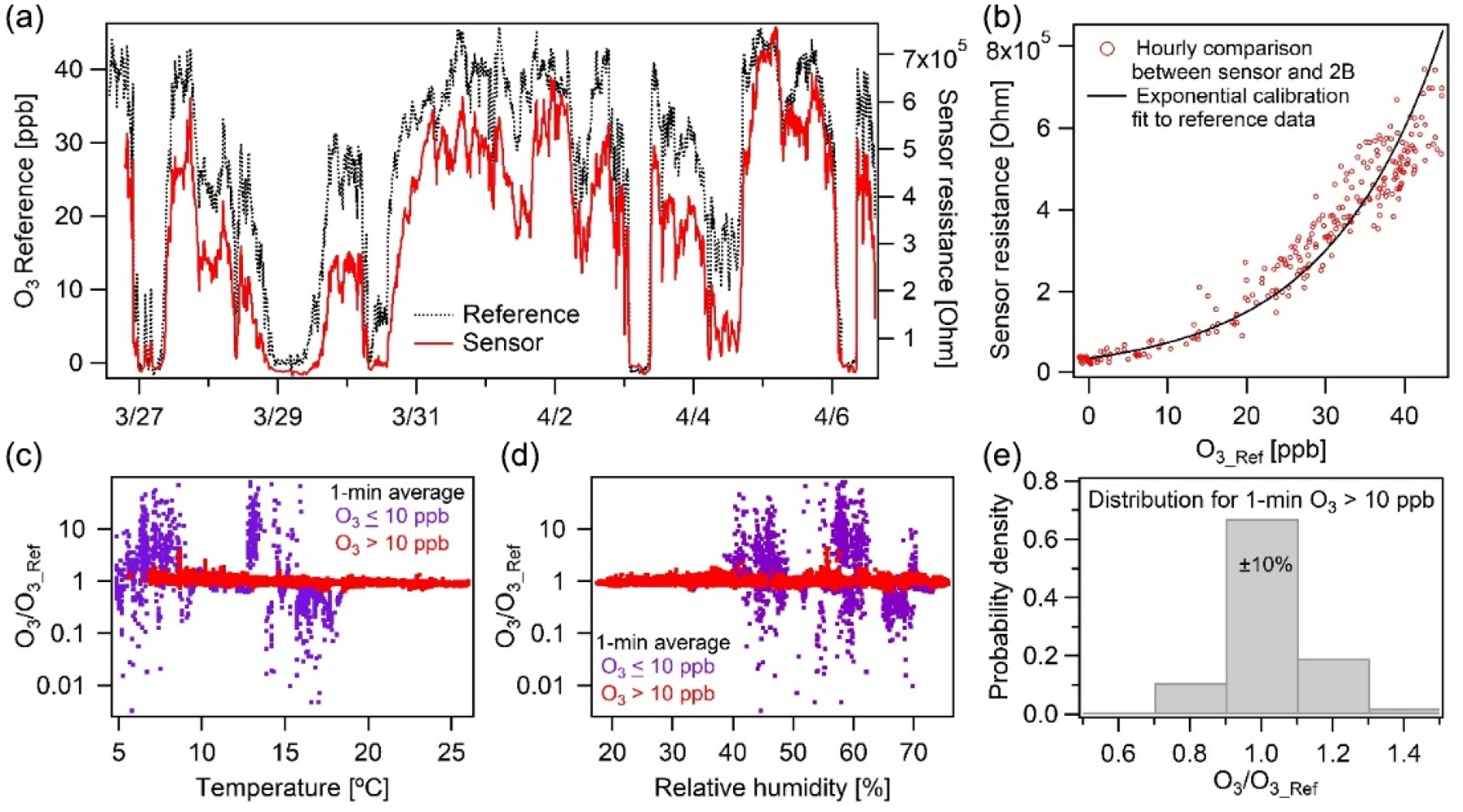
Ozone calibration performance and evaluation (**a**) over 2 weeks in New Haven, CT, shown and (**b**) calibrated against a 2B Tech reference monitor (note panel a shows sensor resistance, not concentration). (**c**–**d**) The ratio of our calibrated vs. reference measurement for concentrations greater and less than 10 ppb over the range of RH and temperatures observed, with no dependence on RH and a slight temperature dependence. (**c**–**d**) At concentrations greater than 10 ppb our measurements are much more accurate, (**e**) with 70% of the 1 min average data falling within ±10%.

The O_3_ sensor exhibited strong correlation (*r* = 0.97*, m* = 0.99) at a high temporal resolution of 1 min in New Haven, CT. The raw sensor resistance followed the ozone concentration measured by the 2B monitor with an exponential relationship (Fig. 6b). With this fitted exponential curve, the ozone concentration from sensor measurement was derived and compared to the 2B monitor results to evaluate the sensor’s performance and dependency on environmental factors (MBE = −0.2 ppb, RMSE = 3.3 ppb). We found that at low ozone concentrations (< 10 ppb), there is considerable measurement discrepancy between the two devices (Fig. 6c and d). This is consistent with the sensor manufacturer’s 10–1000 ppb rating for the device’s measurement range (Table 1). For ozone concentrations higher than 10 ppb, 67% of the data points agree within 10% and 99% within 30% of the reference (Fig. 6e). It is worth noting that the sensor output was not significantly affected by changes in RH during the study, despite large variation in these environmental conditions (Fig. 6d). The temperature effect is more significant with the concentration ratio for our monitor to the reference (for data > 10 ppb), following the relationship 0.85 + 0.70 exp (−0.11*T*). Still, the sensor’s temperature biases are minor here as Fig. 6e presents non-temperature-corrected results.

#### 3.1.6 Nitric oxide

**Figure 7.**
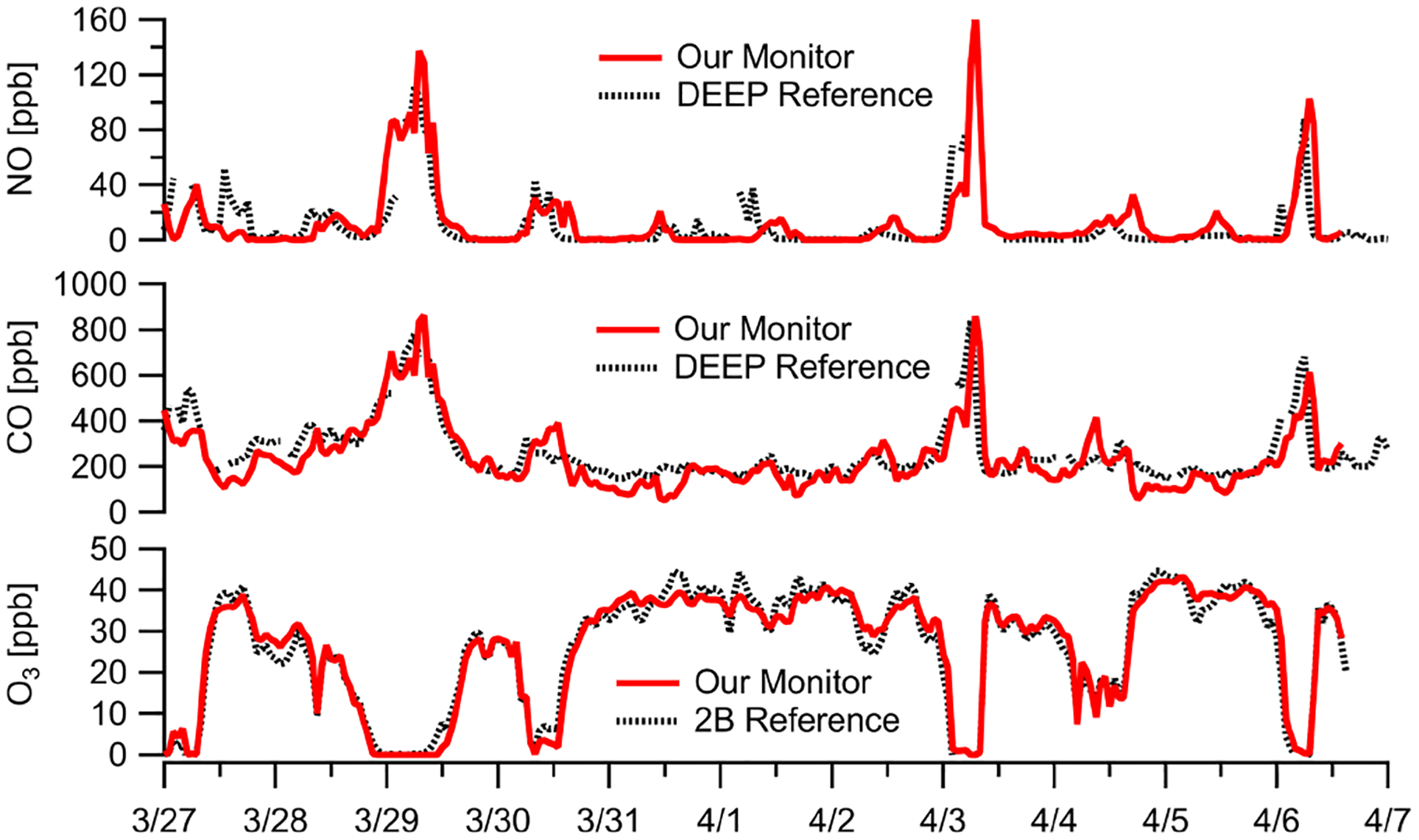
Ambient data at 1 h resolution in New Haven, CT, near construction show large NO enhancements. The presence of NO is confirmed by both the titration of O_3_ (i.e., NO + O_3_ reaction) to zero and large enhancements in CO (a combustion co-pollutant). The near-road DEEP Criscuolo Park site (1.6 km away from sampling location in downtown New Haven) is used for comparison. Note our NO sensor does not have significant cross-response to O_3_ or CO. (See Fig. S11 for additional NO comparisons.) Multipollutant monitor data are corrected for RH–*T* where appropriate.

Without an immediately co-located reference NO monitor, we compared the NO sensor performance against the near-road DEEP Criscuolo Park site (1.6 km away from sampling location in downtown New Haven). The sensor had higher error terms than other low parts-per-billion-level sensors (MBE = +1.6 ppb, RMSE = 16 ppb) likely due to the distance difference and deviations due to local dynamics, but it still showed good agreement (Fig. 7) and reasonable correlation (*r* = 0.74, *m* = 0.86; Fig. S11) at a 1 h resolution. We also leverage O_3_ and CO measurements during the same sampling window to understand NO concentrations as NO readily reacts with O_3_ and is often co-emitted with CO during combustion. The 2-week campaign shows the effect of high NO concentrations on O_3_ abundances due to the reaction NO + O_3_, and CO enhancements coincide with elevated NO concentration levels (Fig. 7). NO concentrations at 1 h resolution during the New Haven deployment range from 0 to 160 ppb with three major concentration enhancements. Each buildup occurred overnight and dissipated around midmorning, potentially owing to periods of decreased ventilation and the accumulation of NO from nighttime traffic emissions in the nocturnal boundary layer without any photochemistry. A 1 h time lag relative to the reference site is observed on some NO spikes, which is also observed in the CO data, and is likely due to differences in sampling location. During the three periods when NO concentrations exceeded 100 ppb for an extended period of time, O_3_ concentrations decreased to ~ 0 ppb and CO concentrations exceeded 600 ppb. It is worth noting that the NO sensor used does not have significant cross-response to O_3_ or CO (Alphasense NO-A4, 2019b).

#### 3.1.7 Others

Other pollutants not discussed in detail here include CH_4_ and SO_2_. The Figaro TGS2600 methane sensor demonstrated high linearity in signal response in the laboratory (Fig. S7) and effective VOC filtration, but a reference monitor was not available and future testing is planned. While the multipollutant monitors field-tested in this paper do not include the SO_2_ sensor, and it is not planned for the SEARCH deployment, our laboratory results (Fig. S6) and past work (Hagan et al., 2018) suggest it is suitable for measurements in locations with SO_2_ concentrations higher than typical urban levels (> 15 ppb).

### 3.2 Portable monitor field testing

**Figure 8.**
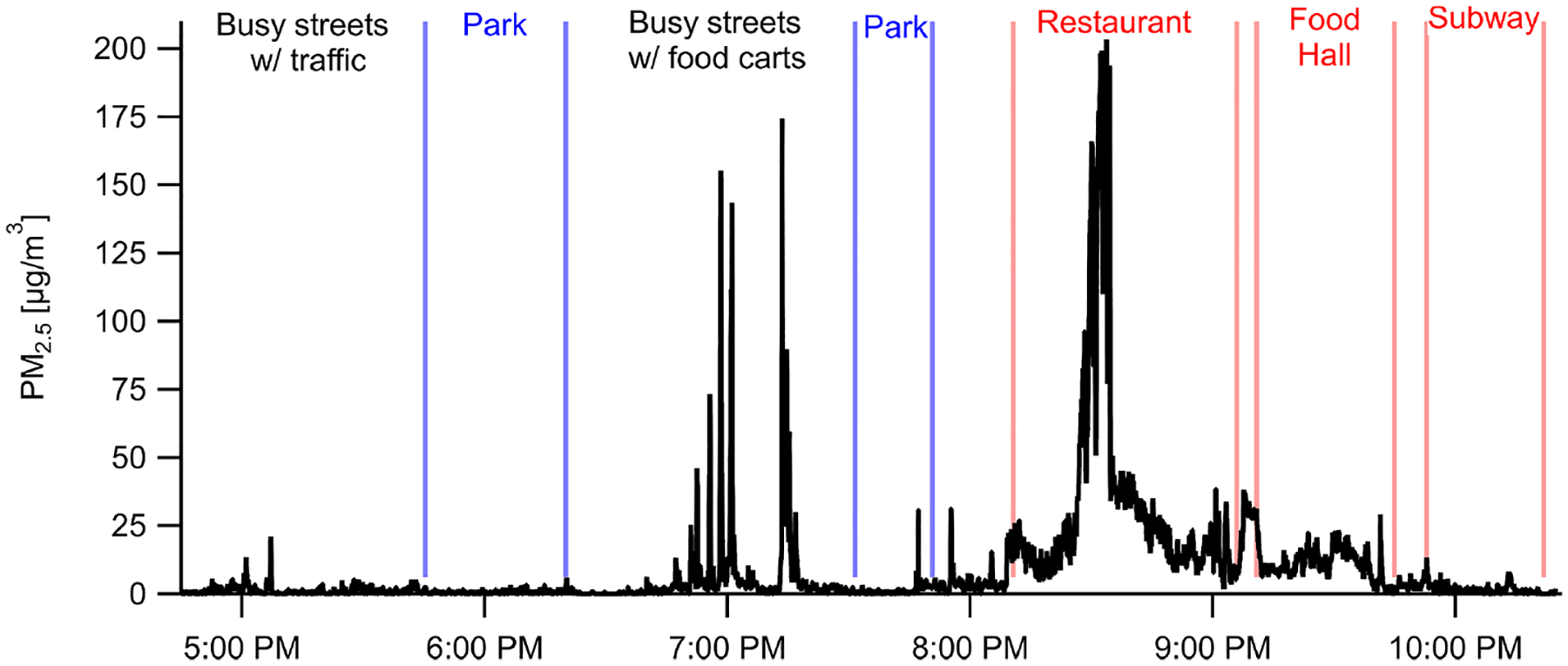
Portable personal monitor data for PM_2.5_ from New York City on 23 June 2018 with labeled events, locations, and nearby sources. Data are shown at 1 s resolution (with RH–*T* correction factors) capturing rapidly changing microenvironments such as emissions from individual food carts (18:45–19:15).

**Figure 9.**
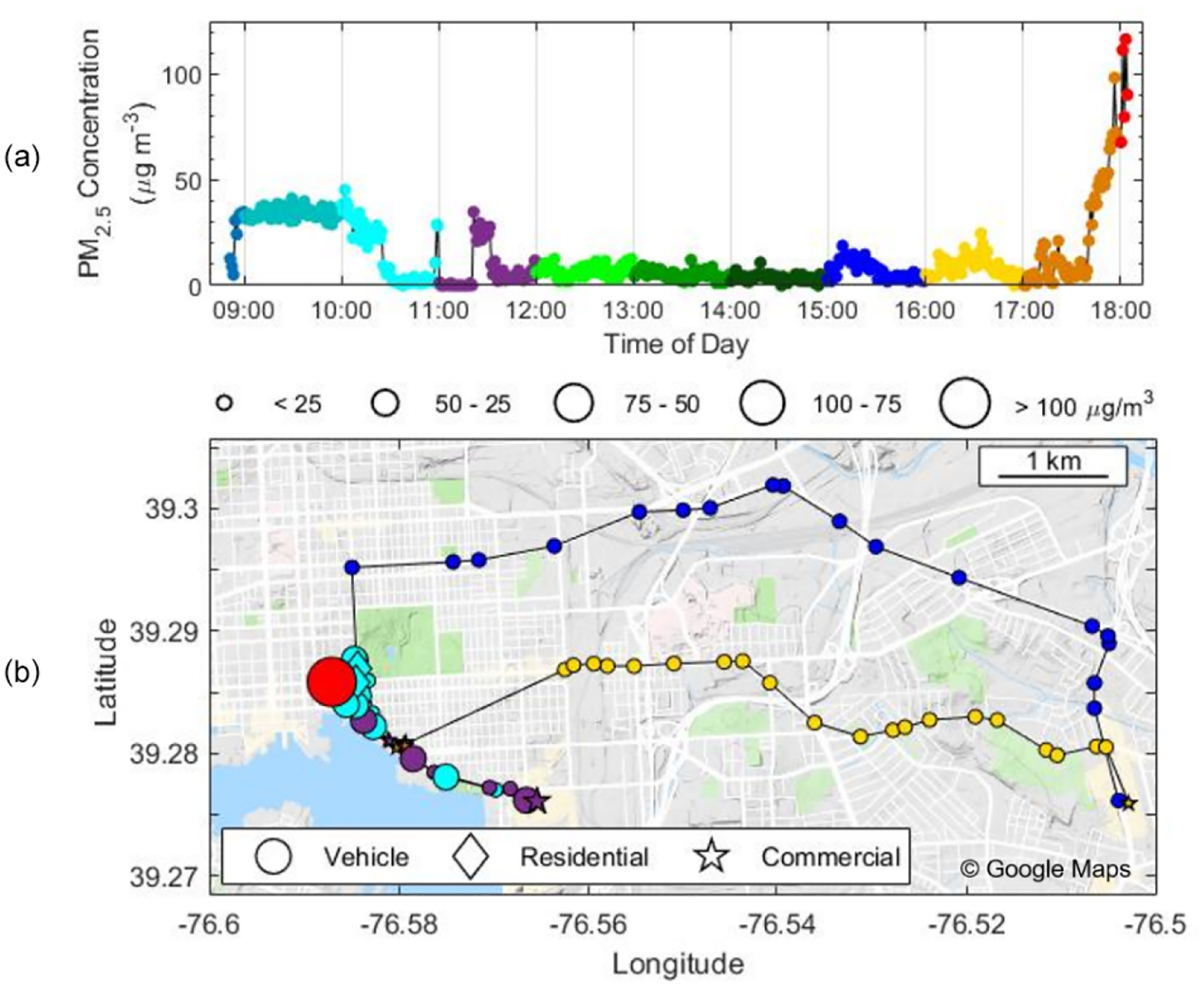
PM_2.5_ mass concentration (μg m^−3^) as a function of (**a**) time and (**b**) mapped with GPS coordinates. The color of the dots in both panels changes each hour to represent the time in (**b**). The background colors in (**a**) and shapes in (**b**) indicate microenvironment, i.e., residential (diamond), commercial (star), and vehicular (circles). The size of the points in (**b**) corresponds to the mass concentration. This test deployment occurred on 2 March 2019.

Personal exposure data were collected using our shoulder-mounted portable monitor in Manhattan, New York City (Fig. 8), and Baltimore, MD (Fig. 9). These results are discussed in brief with a focus on PM_2.5_. In New York City, PM_2.5_ concentrations reached a maximum of 210 μg m^−3^ at a restaurant where the average was 34 μg m^−3^ while inside. Several closely occurring spikes with PM_2.5_ maxima at 30–175 μg m^−3^ occurred in an area with food carts on Broadway Ave. between 34th St. and 57th St., including openflame meat cooking, small power generators, and cigarette smoking. An average concentration of 9 ± 21 μg m^−3^ was encountered across the 6 h period that included a mix of indoor and outdoor environments and a mix of parks and streets. Concentrations while in parks were lower than when walking along streets. The average concentrations across the southern transect of Central Park (17:45; all times in the paper are in EST) and Madison Square Park (19:30) were 0.5 μg m^−3^ and 1.4 μg m^−3^, respectively, compared to 3.9 μg m^−3^ while on the streets before and after. Coupling the high sampling rate of one measurement per second with RH–*T* correction factors, the portable monitor has the ability to capture high-temporal-resolution events (i.e., 10–20 s) for PM_2.5_. PM_1_ had similar trends, with a PM_2.5_*/*PM_1_ ratio of 1.44 at the restaurant, 1.48 at the parks, and 1.44 for the entire study period. Due to the lack of a robust RH–*T* correction factor for PM_1_, we report only the ratio for PM_2.5_*/*PM_1_ in the raw data.

A day-long deployment in Baltimore shows PM_2.5_ concentrations vary widely across locations and transportation modes (Fig. 9) and can be mapped within a city via GPS. Elevated levels of 35 μg m^−3^ occurred in the morning at a restaurant before reaching consistent values of less than 10 μg m^−3^ for most of the day. There were occasional concentration spikes, such as walking through a commercial store (see purple symbols at 11:00) where measurements rapidly rose from ~0 to 30 μg m^−3^. In the afternoon, concentrations spiked to above 110 μg m^−3^ while driving during rush hour with the windows closed. GPS functionality was maintained throughout the study and accurately depicted the path of the participant, yet some path information in Fig. 9 is not depicted during vehicle transport due to averaging to 1 min intervals.

### 3.3 Sensor response time

**Figure 10.**
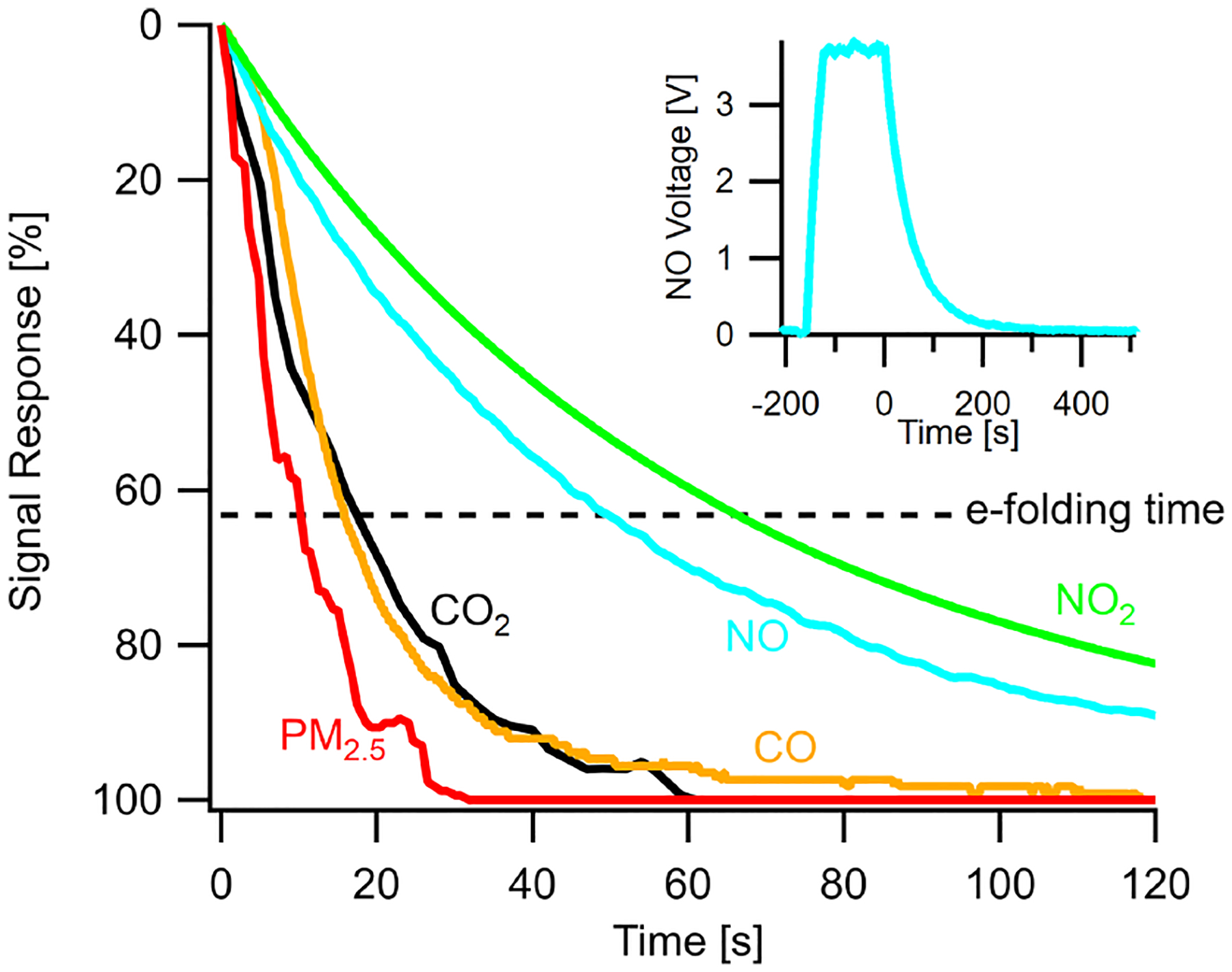
Instrument response times shown as the normalized signal response of various sensors in the stationary monitor to a step change in target pollutant concentration and their respective *e*-folding times. Insert shows a typical step change of calibration gas and the sensor response. Time zero indicates when the multipollutant monitor switched to sampling zero air after reaching a steady-state response to the pollutant. Gas sensor response times vary due to the individual sensors’ diffusive or electrochemical timescales.

A key performance characteristic of any field-deployed analytical instrument is its response time to changes in pollutant concentrations, especially in dynamic urban environments where concentrations change rapidly with source proximity or microenvironments. This response time is a function of air exchange rates within the sampling system and the individual sensor response times, which are inherently limited in some sensors involving electrochemical processes. A useful metric to examine this is the *e*-folding time (i.e., a decrease to a signal of 1 – 1/*e* or ~63%) of sensor signals due to abrupt changes in pollutant concentration. A long *e*-folding time indicates a sluggish sensor, while a short *e*-folding time indicates a responsive sensor that can respond to a dynamic environment and distinguish changes at higher temporal resolution. To characterize the sample delivery systems, Fig. 10 shows the response of several sensors in the multipollutant monitor and their *e*-folding times. PM_2.5_ has the shortest *e*-folding time of roughly 10 s (PM_1_ and PM_10_ are similar) due to its optical sensing technique (in a separate sampling inlet). CO and CO_2_ have similar *e*-folding times of 20 s, demonstrating an ability to capture changes at under 1 min resolution (see Fig. S10 for analysis of 20 s PM_1_ and CO roadside plumes). NO and NO_2_ take longer to respond, 50 and 65 s respectively, but are still capable of capturing urban dynamics at a 5–10 min resolution.

### 3.4 Online calibration processes

#### 3.4.1 Standard gas cylinder delivery

**Figure 11.**
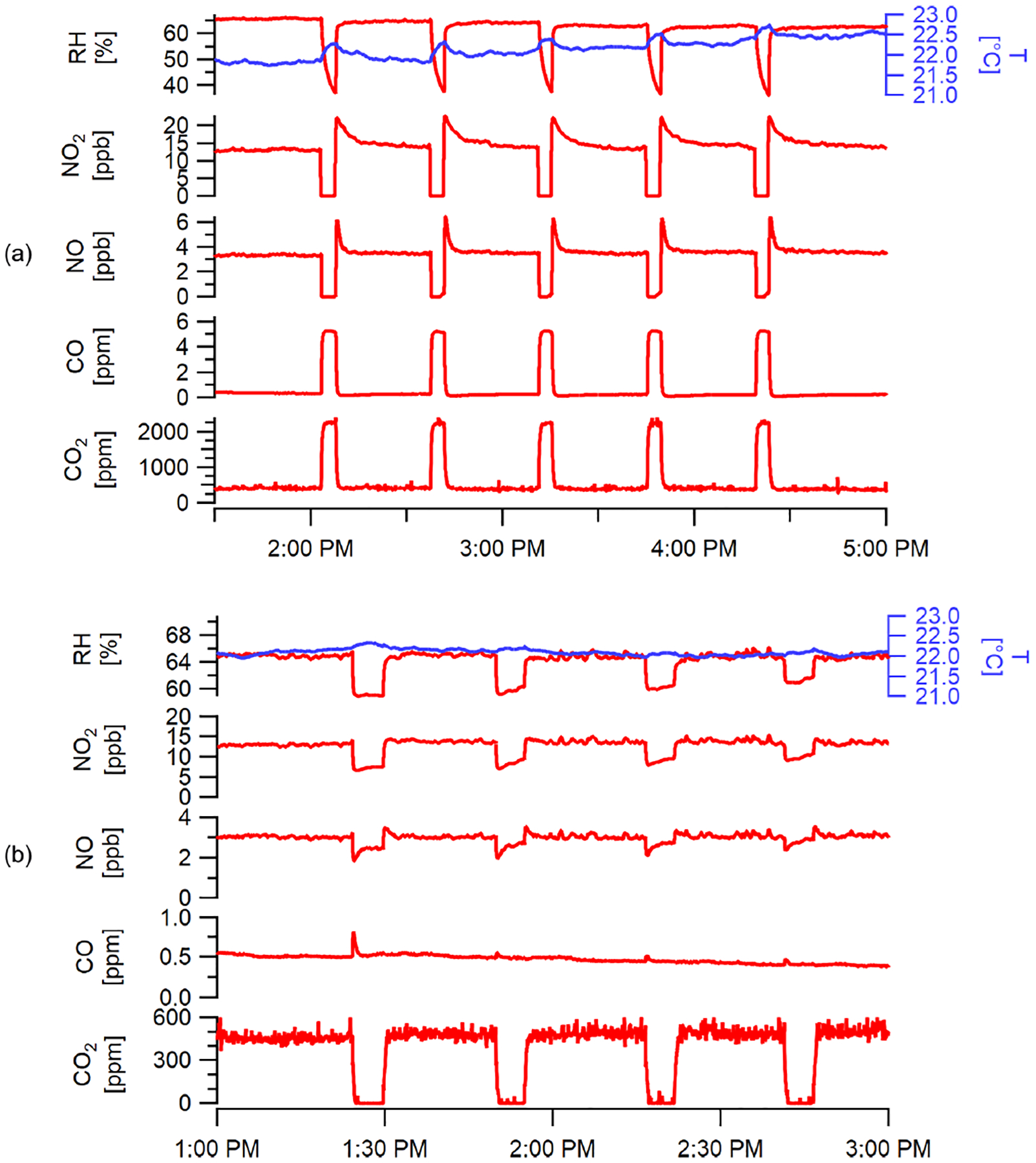
Performance of the onboard calibration system over sequential cycles (for demonstration) showing (**a**) the span gas delivery for CO and CO_2_ (and zero air to provide zero concentrations for NO and NO_2_) over five repeated cycles and (**b**) the zeroing function for CO_2_ over four cycles where other signals are shown to illustrate minimal changes with zero trap (NO_*x*_ changes are due to shown RH changes). PM zeroing function results are shown in Fig. S13.

A laboratory test of the gas cylinder delivery system is shown in Fig. 11a delivering known concentrations of CO and CO_2_ in a balance of nitrogen for a span check as well as zero concentration signals for other sensors (e.g., NO_2_, NO). With the small internal volume of the manifold flushed by 30 sccm of standard gas, it quickly reaches stabilized signals within the 3 min calibration periods, consistent with expectations based on Fig. 10. Five repeated runs at a delivery pressure of 35 psig (prior to the constriction tubing that substantially reduces pressure to near 1 atm) demonstrated consistent behavior and is representative of initial performance in field-tested units. Expected shifts in RH are observed (and can be used in field to evaluate RH sensitivity), but the permeation device maintains humidity at relevant conditions despite a completely dry standard gas.

#### 3.4.2 Zero trap

Figure 11b shows the effective removal of CO_2_ using the gas-zeroing function, while stable concentrations are observed for non-zeroed gases. Changes in NO concentrations (and less so for NO_2_) occur due to the observed changes in RH. During long-term field deployment, RH changes occurring during the use of both the calibration gas cylinder and the zero trap are also useful indicators to check in-field RH-dependent changes in response factors and zero signals, respectively, when occurring under stable concentrations. Similarly, differences in temperature between zeroing periods can be used to check temperature-related variations for sensors with significant temperature response. Figure 11b does not include O_3_ data as the levels in the laboratory were too low for sensor response to move off the baseline. Experiments at higher concentrations indicate that stainless steel is an effective scavenger of O_3_ (see Fig. S12).

#### 3.4.3 PM zero

Field testing of the PM zero method was conducted near food cart vendors in New Haven, CT. Ambient concentrations ranged between 3 and 48 μg m^−3^ with rapid changes due to moderate wind levels and proximity to active sources. After switching the valves, PM levels were effectively zeroed for 2 min and rose back immediately to higher concentrations and saw large spikes after the zero ended (see Fig. S13). For monitors in very close proximity to highly concentrated plumes (e.g., > 250 μg m^−3^), longer zero periods or scheduling during low-activity periods may be necessary to fully flush the inlet and avoid/isolate bias from concentrated plumes.

### 3.5 Comparison with literature

To contextualize the performance of our monitors, Tables 3 (for NO_2_, NO, and CO) and 4 (for CO_2_, O_3_, and PM_2.5_) show a summary of co-location statistical data with several recent literature field deployments. For a more extensive comparison, see Karagulian et al. (2019). The presented performance metrics are specific to the region and conditions they were evaluated in, and differences in sampling locations, environmental conditions, pollutant mixtures, and testing durations should be considered in future applications. For NO_2_, our multipollutant monitor had a higher *r*^2^ (0.77) than other studies except for Bigi et al. (2018) (*r*^2^ = 0.80), with our MAE and RMSE lower by 2.1 and 3 ppb, respectively. The NO sensor shows lower correlation than other studies such as Cross et al. (2017) in terms of *r*^2^ (their *r*^2^ of 0.84 compared to our 0.54) and error terms (their RMSE of 4.52 ppb compared to our 16 ppb), though we are comparing against a reference instrument located 1.6 km away with evident localized dynamics (see Sect. 3.1.6). For CO, the multipollutant monitor performed similarly to other studies using Alphasense electrochemical sensors, such as Zimmerman et al. (2018), where our *r*^2^ and MAE were lower by 0.11 and 3 ppb, respectively. To our knowledge this work is the first low-cost urban air sensor network implementation of the Alphasense IRC-A1 sensor. Zimmerman et al. (2018) measured CO_2_ using an SST Sensing Ltd CO2S-A, but no reference instrument was available for their co-location study. One study that did report CO_2_ co-location results was Spinelle et al. (2017), which showed higher *r*^2^ (0.51–0.79) but similar slope values after using an artificial neural network for calibration. Shusterman et al. (2016), as a part of the BErkeley Atmospheric CO_2_ Observation Network implement a more expensive, yet still lower cost than reference instrumentation, Vaisala CARBOCAP GMP343 with extremely high correlation (*r*^2^ = 0.999) during a 5 d comparison. For O_3_, the deployments used for comparison primarily utilized a form of the Alphasense Ox-B4 sensor (in tandem with NO_2_ electrochemical sensors). Our MiCS sensor performed well compared to other co-locations in regards to *r*^2^ and *m* values, with only Zimmerman et al. (2018) (using the updated Ox-B431, which is similar to the A431 which we employ in our portable monitors) reporting similar MBE. Ripoll et al. (2019) used both an Alphasense Ox-B431 and MiCS-2614 sensor and found that both exhibited strong performance, similar to our results. For PM_2.5_, we compare our sensors primarily with selected results from AQ-SPEC testing (i.e., five highest *r*^2^ values) from Feenstra et al. (2019). PM_2.5_ sensors saw a wide range of *r*^2^ values being reported (0.73–0.95) with the highest coming from the PurpleAir PA-II which uses a Plantower sensor and the two next highest being Plantower A003 sensors from this study. To summarize the performance distribution of our pollutant measurements, Fig. S14 compares the values from Tables 3 and 4 to the “best selection region” defined by Karagulian et al. (2019).

### 3.6 Best practices

The field of low-cost air quality sensors is rapidly improving, and new generations of monitoring devices should strive to further improve accuracy and precision. Here we present a list of lessons learned, obstacles faced, and recommendations for future design, fabrication, and deployment. Careful consideration of electronic design and sensor selection can eliminate complications later in the process. We make the following recommendations: choose low-noise components where applicable to enhance precision and improve detection limits, transform signals from analog to digital close to the sensor to preserve signal, utilize ADCs of sufficiently high resolution for the application to achieve the resolution necessary for the pollutant measurement application, use electronic shielding on sensitive sensing or signal transmission components, monitor power delivery in real time and report back auxiliary and supply signals, and measure RH–*T* -dependent channels where applicable (e.g., auxiliary electrode on Alphasense sensors).

Good design should take into account sensor-to-sensor performance and practice good quality assurance and quality control (QA/QC) of sensors, both prior to installation and for a trial period after installation. For some of the sensors used in the multipollutant monitor, there was more deviation from lot to lot than anticipated, which requires careful laboratory calibration to correct. To minimize the impact of sensor variability on field measurements, characterize sensors before installation and deployment for supplemental quality control purposes. If possible, compare new sensors against typical response patterns found in the deployment already in order to gauge whether a sensor or circuit board may be malfunctioning early on. With a large network, some amount of automation will be necessary to quickly determine malfunctioning monitors. Also, carefully consider the position within the manifold and implement measures to keep the sensors clean and away from interferences, such as upstream particle filtration for gas-phase sensors or positioning the NDIR CO_2_ sensor last to reduce the influence of waste heat, respectively.

Active flow is critical and allows us to achieve high-temporal-resolution measurements with *e*-folding times below 1 min for most pollutants. In urban settings and for personal exposure studies this provides additional data points to identify rapidly changing emissions and environmental conditions. When deploying the monitors, use a watertight enclosure with inlets pointed downwards to avoid light and water intrusion, shade them from direct sunlight if possible at a given site to reduce temperature swings that exacerbate temperature-dependent calibration changes, implement a mesh covering to the inlets to prevent insects from entering, and be mindful of point source emissions nearby to reduce undesired bias. Additionally, check for flow balance consistently during construction and then periodically in the field (during deployment or servicing) to (i) confirm high active flow rates, (ii) verify that measurements are not being compromised with air from within the enclosure, and (iii) ensure that onboard calibrations are performed properly. Additionally, in order to better assess issues remotely, choice of wireless connectivity should be evaluated. Cell connections are convenient and allow for the monitor to be placed nearly anywhere but can be spotty at times and can be more expensive to operate than a WiFi-based approach. Protocols for remote access into the monitors to address problems and prompt a restart are desirable to minimize wasted travel time to sites.

## 4 Conclusions

Our multipollutant monitors advance the state of the field by monitoring nine or more gas and size-resolved PM pollutant data streams simultaneously in an optimized fast-response active-flow system. The stationary monitor includes a novel on-board calibration system and the portable shoulder-mountable monitor samples in the breathing zone. We implement low-noise electronic design, GPS tracking, and cellular communications to communicate ambient and calibration data in real time – all to enable more accurate and precise cost-effective monitor networks for stationary or mobile platforms. The calibration system is flexible and can be adjusted for a variety of analytes of interest via tuning of the calibration gas or zero trap. Additionally, a greater range of RH–*T* points can be gained by increasing calibration frequency with strategic timing of calibration functions across the day. Still, there is a need for continual long-term evaluation and improvement of laboratory and field calibration procedures, effects from RH–*T*, and comparisons to reference instrumentation through permanent and temporary co-location. With most pollutants achieving high correlation in urban field evaluations, these systems are ready for large-scale network deployments and smaller-scale targeted measurements.

## Supplementary Material

SI

## Figures and Tables

**Table 1.** Technical specifications concerning the pollutant and environmental sensors tested and used.

Sensor target	Part number	Method	Observed LOD	Usage^c^
CO	Alphasense CO-A4	Four-electrode electrochemical	20 ppb	S, P
NO	Alphasense NO-A4	Four-electrode electrochemical	3 ppb	S
NO_2_	Alphasense NO2-A43F	Four-electrode electrochemical	1 ppb	S, P
SO_2_	Alphasense SO2-A4	Four-electrode electrochemical	15 ppb	–
O_3_	Alphasense OX-A431	Four-electrode electrochemical	1 ppb	P
CO_2_	Alphasense IRC-A1	Infrared, pyroelectric	^a^	S, P
CH_4_	Figaro TGS 2600	Metal oxide resistance	^a^	S
O_3_	MiCS-2614	Metal oxide resistance	10 ppb	S
PM_1_, PM_2.5_, PM_10_	Plantower A003	Optical particle counter	1 μg m^−3^	S, P
RH & *T*	Sensirion SHT25	–	0%–100%, 0–120 °C^b^	S, P

a Below ambient background concentrations.

b From manufacturer data sheet.

c Used in the stationary (S) and/or portable (P) version of the multipollutant monitor.

**Table 2.** Lab calibration procedure and environmental condition ranges for gases. Each non-zero gas concentration is maintained for 90 min.

Gas	Concentrations/ranges
NO	0, 5, 10, 30, 50 ppb
CO	0, 1, 2, 3, 4 ppm
O_3_	10, 20, 50, 75, 100 ppb*
NO_2_	0, 5, 10, 30, 50 ppb
CH_4_	1, 1.5, 2, 2.5 ppm
CO_2_	400, 500, 700 ppm
RH	5%–85%
*T*	20–40 °C

* Denotes that each concentration is repeated.

**Table 3.** Summary of statistics and comparison of the SEARCH stationary multipollutant monitor to several recent literature field studies (NO_2_, NO, and CO). Dashes indicate data not reported or readily accessible for a given metric.

Pollutant	Sensor type	Temp. res.	Deployment length	Deployment location	Unit	*m*	*r*	*r* ^2^	MBE	MAE	RMSE	Reference
NO_2_	Alphasense NO2-B4	10 min	4 months	Zurich-Kaserne, Switzerland	ppb	–	–	0.80	–	5.8	8.3	Bigi et al. (2018) ^b^
	Alphasense NO2-B43F	15 min	24 d	Pittsburgh, PA	ppb	0.64	–	0.67	−0.4	3.48	–	Zimmerman et al. (2018)
	Alphasense NO2-B43F	5 min	89 d	Boston, MA	ppb	0.81	–	0.69	1.2	3.45	4.56	Cross et al. (2017)
	Alphasense NO2-B4	15 min	3 months	Oslo, Norway	ppb	0.38^a^	0.49	–	13.3	26.23	30.27	Castell et al. (2017)
	Alphasense NO2-B42F/B43F	1 h	1 week	Oakland, CA	ppb	–	0.61	–	–	4.12	–	Kim et al. (2018)
	Alphasense NO2-A43F	1 h	4 weeks	Baltimore, MD	ppb	0.93	0.88	0.77	0.8	3.7	5.3	This work
NO	Alphasense NO-B4	10 min	4 months	Zurich-Kaserne, Switzerland	ppb	–	–	0.94	–	2.26	3.54	Bigi et al. (2018) ^b^
	Alphasense NO-B4	5 min	–	Boston, MA	ppb	0.94	–	0.84	0.97	2.83	4.52	Cross et al. (2017)
	Alphasense NO-B4	15 min	3 months	Oslo, Norway	ppb	0.93^a^	–	0.86	−0.54	12.48	16.35	Castell et al. (2017)
	Alphasense NO-B4	1 h	1 week	Oakland, CA	ppb	–	0.88	–	–	3.63	–	Kim et al. (2018)
	Alphasense NO-A4	1 h	2 weeks	New Haven, CT	ppb	0.86	0.74	0.54	1.6	8.5	16	This work
CO	Alphasense CO-B4	15 min	41 d	Pittsburgh, PA	ppb	0.86	–	0.91	0.1	38	–	Zimmerman et al. (2018)
	Alphasense CO-B4	5 min	75 d	Boston, MA	ppb	0.94	–	0.88	−10.4	24.8	32.9	Cross et al. (2017)
	Alphasense CO-B4	15 min	3 months	Oslo, Norway	ppb	0.88^a^	0.6	–	−147.21	149.35	170.99	Castell et al. (2017)
	Alphasense CO-B4	1 h	1 week	Oakland, CA	ppb	–	0.74	–	–	50.93	–	Kim et al. (2018)
	Alphasense CO-A4	10 min	1 week	New Haven, CT	ppb	1.2	0.92	0.84	5	41	59	This work
	Alphasense CO-A4	1 h	1 week	New Haven, CT	ppb	1.2	0.96	0.92	6	33	43	This work

a Slope and intercept value from unit 688150 in Table 2.

b Taken from Fig. 11 from SU009.

**Table 4.** Summary of statistics and comparison of the SEARCH stationary multipollutant monitor to several recent literature field studies (CO_2_, O_3_, and PM_2.5_). Dashes indicate data not reported or readily accessible for a given metric.

Pollutant	Sensor type	Temp. res.	Deployment length	Deployment location	Unit	*m*	*r*	*r* ^2^	MBE	MAE	RMSE	Reference
CO_2_	Gascard NG & S-100H	1 h	85–87d	Ispra, Italy	ppm	0.48–0.67	–	0.51–0.79	–	–	–	Spinelle et al. (2017) ^b^
	Vaisala CARBOCAP GMP343	1 min	2 weeks	Oakland, CA	–	–	–	0.999	–	–	–	Shusterman et al. (2016)
	Alphasense IRC-A1	1 h	3 weeks	Baltimore, MD	ppm	0.59	0.66	0.44	3.4	8.7	11	This work
O_3_	Alphasense Ox-B431	15 min	38 d	Pittsburgh, PA	ppb	0.82	–	0.86	−0.14	3.36	–	Zimmerman et al. (2018)
	Alphasense Ox-B421	5 min	87 d	Boston, MA	ppb	0.47	–	0.39	0.78	7.34	9.71	Cross et al. (2017)
	Alphasense Ox-B421	15 min	3 months	Oslo, Norway	ppb	0.26^a^	0.54	–	6.76	19.87	22.2	Castell et al. (2017)
	Alphasense Ox-B431	1 h	5 months	Piacenza, Italy	ppb	0.89	–	0.88	–	–	–	Ripoll et al. (2019)
	MiCS-2614	1 h	5 months	Piacenza, Italy	ppb	0.90	–	0.89	–	–	–	Ripoll et al. (2019)
	Alphasense Ox-B431/B421	1 h	1 week	Oakland, CA	ppb	–	0.69	–	–	5.04	–	Kim et al. (2018)
	MiCS-2614	1 min	1.5 weeks	New Haven, CT	ppb	0.99	0.97	0.94	−0.2	2.7	3.3	This work
	MiCS-2614	1 h	1.5 weeks	New Haven, CT	ppb	1.01	0.98	0.96	−0.2	2.4	2.9	This work
PM_2.5_	TSI AirAssure	1 h	8 weeks	Riverside, CA	μg m^−3^	1.06	–	0.73	3.9	5.6	7.8	Feenstra et al. (2019) ^c^
	SainSmart P3 Plantower PMS5003	1 h	8 weeks	Riverside, CA	μg m^−3^	1.48	–	0.76	3.9	5.4	7.8	Feenstra et al. (2019) ^c^
	Aeroqual AQY Nova SDS011	1 h	8 weeks	Riverside, CA	μg m^−3^	0.98	–	0.79	−3.1	4.6	6.1	Feenstra et al. (2019) ^c^
	Shinyei PM Eval. Kit PPD71	1 h	8 weeks	Riverside, CA	μg m^−3^	1.11	–	0.74	0.2	4.4	6.4	Feenstra et al. (2019) ^c^
	PurpleAir PA-II Plantower PMS5003	1 h	8 weeks	Riverside, CA	μg m^−3^	1.63	–	0.95	4.8	6.8	10.1	Feenstra et al. (2019) ^c^
	AQMesh OPC v3.0	15 min	3 months	Oslo, Norway	μg m^−3^	–	0.51	–	−0.03	3.08	5.57	Castell et al. (2017)
	Plantower A003	1 h	4 weeks	Baltimore, MD	μg m^−3^	1.0	0.91	0.82	0.9	3.1	4.3	This work

a Slope and intercept value from unit 688150 in Table 2.

b From correlation using an artificial neural network calibration.

c Top five performing (by *r*^2^) monitors.
