# Supplementary material for: Stationary and portable multipollutant monitors for high-spatiotemporal-resolution air quality studies including online calibration
Source: Atmos Meas Tech. Author manuscript; Available in PMC 2022 May 6. (PMC9074123; doi:10.5194/amt-14-995-2021)
Supplement: SI [file NIHMS1802124-supplement-SI.pdf]

Supplement of Atmos. Meas. Tech., 14, 995–1013, 2021
https://doi.org/10.5194/amt-14-995-2021-supplement
© Author(s) 2021. This work is distributed under
the Creative Commons Attribution 4.0 License.

Atmospheric Measurement Techniques
Open Access

Supplement of

Stationary and portable multipollutant monitors for high-spatiotemporal-resolution air quality studies including online calibration

Colby Buehler et al.

Correspondence to: Drew R. Gentner (drew.gentner@yale.edu)

The copyright of individual parts of the supplement might differ from the CC BY 4.0 License.

Table of Contents: Supporting Figures and Tables

List of Tables

Table S1: Physical and electronic technical information on the stationary and portable monitors. 4

List of Figures

Figure S1: Voltage to concentration conversion. 2
Figure S2: Environmental correction factors 3
Figure S3: Photos of the stationary multipollutant monitor. 4
Figure S4: Calibration system flow diagram 5
Figure S5: Data management and visualization software 6
Figure S6: Demonstration of linearity of electrochemical sensor calibration 7
Figure S7: Calibration data for the Figaro TGS 2600 CH_4 sensor 7
Figure S8: PM sensor intercomparison (raw data) for 5 co-located monitors 8
Figure S9: CO comparisons to reference instrument for high and low temperature readings 8
Figure S10: Fast monitor response times to concentration changes 9
Figure S11: NO sensor regression and probability density 9
Figure S12: Performance of the zero trap for O_3 removal 10
Figure S13: $PM_{2.5}$ and PM_{10} sensor zero 10
Figure S14: Sensor r^2 and slope values for co-location data 11

S1 Concentration Conversion Methodology and Example

First, raw voltages are collected and converted to concentrations based on a laboratory derived calibration curve for each box. For the electrochemical sensors, we use the differential voltage, which is the working electrode minus the auxiliary electrode.

$$Volt_{diff} = Volt_{WE} - Volt_{AE}, \tag{S1}$$

In the case below for this particular monitor there is a linear relationship between the differential voltage and the known concentration of reference gas (see Fig.S1). The fit follows the form of:

$$C_{raw} = a_{cal}\, Volt_{diff} + b_{cal}, \tag{S2}$$

where C_{raw} represents the non-RH/T corrected concentration value. This fit works well for the Alphasense electrochemical sensors. See notes at the end for CO_2 sensor fits. The limit of detection values shown in Table 1 are calculated by taking three times the standard deviation of the sensor output in zero air and dividing by the slope (a_{cal}).

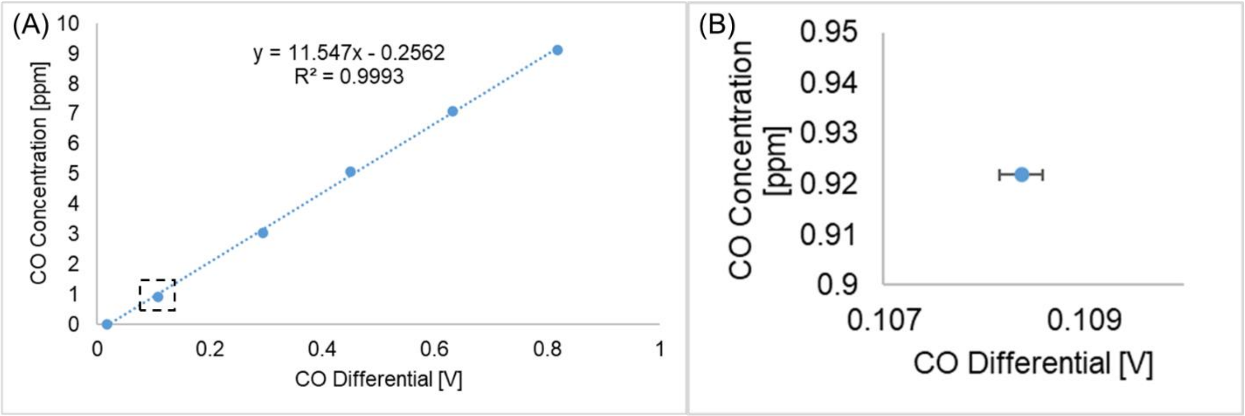

Figure S1: (**a**) Laboratory linear calibration of one CO sensor under a range of known gas concentrations and (**b**) zoom in on one point to show minimal standard deviation in differential signal for a several minute period under constant laboratory RH/T setting.

For this study we implement RH/T correction factors from co-location evaluations rather than from lab calibrations. Using a training period, we apply the following equations to reach a final concentration:

$$C_T = \frac{C_{raw}}{a_T T^2 + b_T T + c_T}, \tag{S3}$$

$$C_{RH/T} = \frac{C_T}{a_{RH} RH^2 + b_{RH} RH + c_{RH}}, \tag{S4}$$

First the ratio of the monitor value over the reference is plotted against temperature. We apply a best-fit linear and quadratic regression to determine the relationship (see Fig. S2). Then we examine the r^2 value for each and choose the fit with the strongest correlation. Here, the best temperature relationship is determined to be quadratic (apply fit to Eqn. S2 to extract temperature corrected concentration). Then the temperature corrected concentration relative to reference is plotted against

relative humidity. Here, the best relative humidity relationship is linear (apply fit to Eqn. S3 to extract the final concentration data). With these two relationships we can apply the RH/T correction factors to the rest of the data.

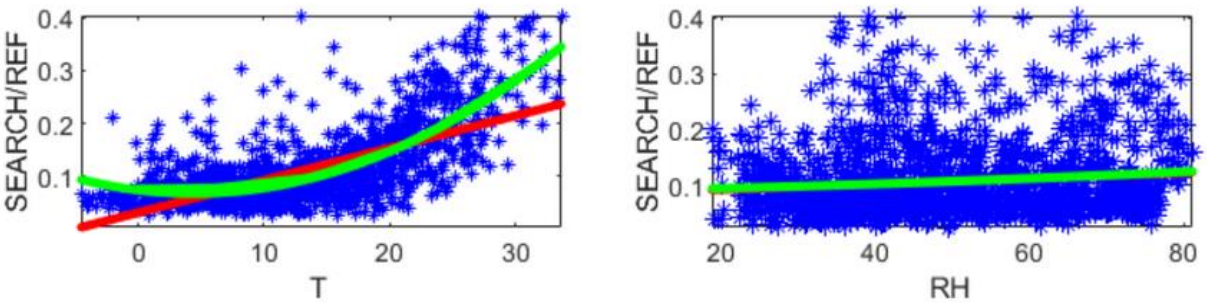

Figure S2: Training period example for the NO_2 sensor after a one-week co-location. The left shows the ratio of the multipollutant monitor concentration relative to the reference monitor against T. The right shows the ratio of the temperature corrected multipollutant monitor concentration relative to the reference against RH. These plots are used to generate the coefficients for the denominators of Eqn S3 and Eqn S4.

Notes for CO_2: The CO_2 calibrations follows Alphasense technical documentation rather than the above methodology (Alphasense Ltd., 2014). No RH adjustment is made for the CO_2 sensor, only the Alphasense-specified T correction. In addition, when aggregating the data to evaluate the CO_2 sensor relative to a reference, we implement a rolling geometric mean rather than arithmetic for averaging and smoothing sensor response. All other sensors use an arithmetic mean for calculations.

S2 Comparison with Literature

In a systematic review of the literature, Karagulian et al. (2019) compiled the r^2 and slope values of numerous field and laboratory co-locations for low cost monitors. They then determined a “best selection region” for monitors from the union of high correlation (r^2>0.75) and minimal over or under estimation (0.5<m<1.5). Three out of our five co-located pollutants (O_3, CO, $PM_{2.5}$) discussed in detail lie inside of the best selection region, with one additional pollutant (NO_2) along the edge (Fig. S12). Two pollutants do not fall near this region (CO_2 and NO) for reasons previously discussed in the main text.

References:

Alphasense Ltd.: AAN 201-06 NDIR: Gas Concentration Calculation Overview, [online] Available from: http://www.alphasense.com/WEB1213/wp-content/uploads/2014/12/AAN_201-06.pdf, 2014.

Karagulian, F., Barbiere, M., Kotsev, A., Spinelle, L., Gerboles, M., Lagler, F., Redon, N., Crunaire, S. and Borowiak, A.: Review of the performance of low-cost sensors for air quality monitoring, Atmosphere (Basel)., 10(9), doi:10.3390/atmos10090506, 2019.

Table S1: Physical and electronic technical information on the stationary and portable monitors.

	Stationary	Portable
Dimensions (cm)	28 x 18 x 14	15 x 6.5 x 5[1]<br>23 x 12 x 6.5[2]
Power	Wall Outlet	Tenergy #31800 Rechargable Li-ion Battery (11.1 V 10.4 Ah, 115.45 Wh)
Data Storage	Onboard 16 GB SanDisk Edge MicroSD	
Data Transmission	Cellular communication to InfluxDB via Telit LE910C1-NS module at 10s interval	N/A
Microcontroller	Cypress 68 pin PSoC 5lp	
Software	PSoC Creator 4.1	
Cell Antenna	LTE + Cellular flexiiANT Mitis SRFL026	N/A
GPS Antenna	N/A	Octaband LTE Cellular Embedded antenna (FPC) 1002292
Operational Duration	Indefinitely	~3 days per single charge

[1]Dimensions for shoulder mountable sensor manifold and enclosure

[2]Dimensions for motherboard enclosure and battery

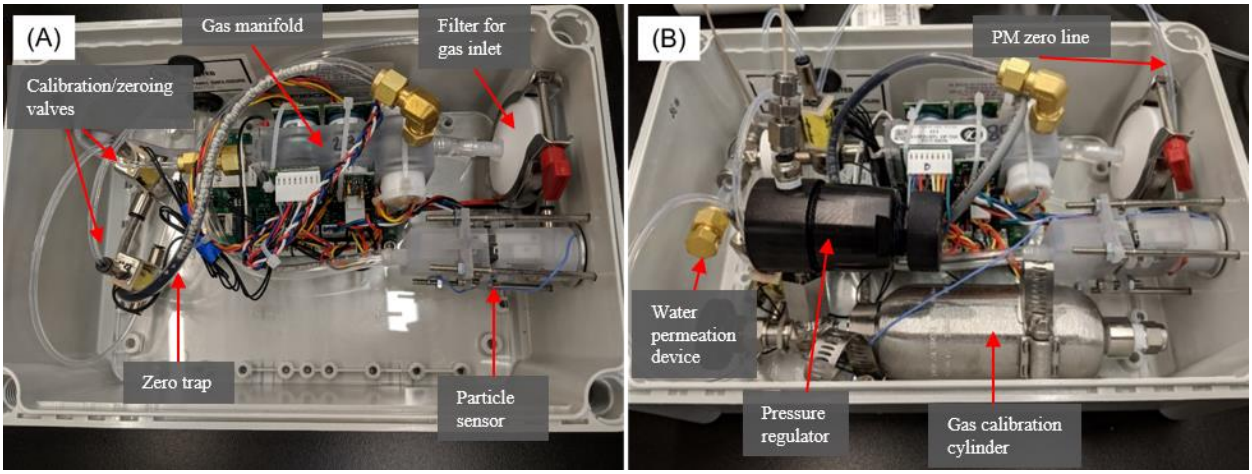

Figure S3: Photo of the (**a**) non-cylinder and (**b**) cylinder version of the stationary multipollutant monitor.

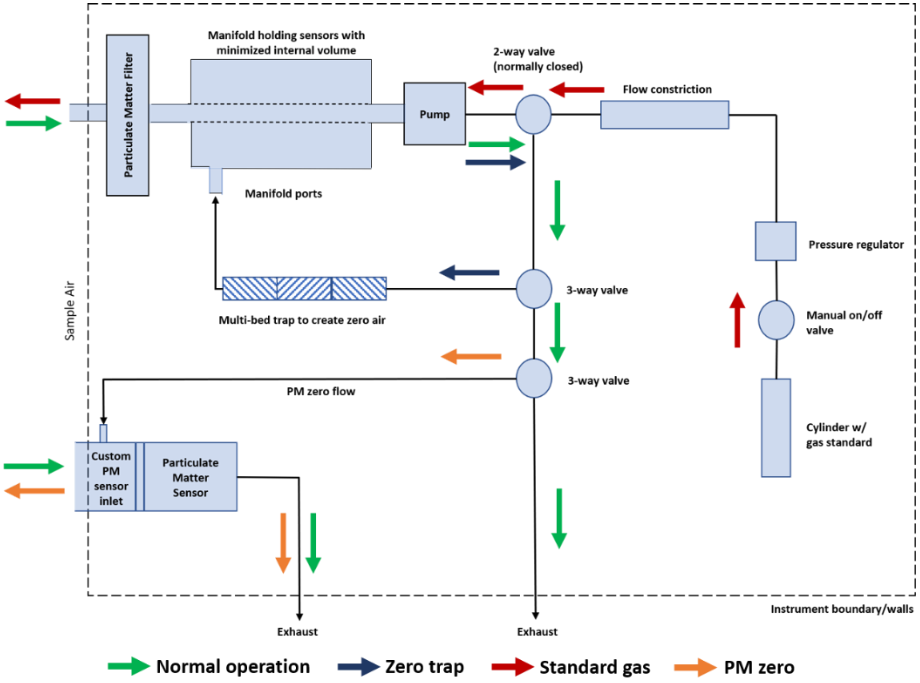

Figure S4: Calibration system flow diagram in the overall system schematic for Yale multipollutant monitor. This system includes calibration approaches for both the gas-phase sensors and particulate matter sensor. The pump operates during all sampling modes except when the standard gas cylinder is used.

(A)

(B)

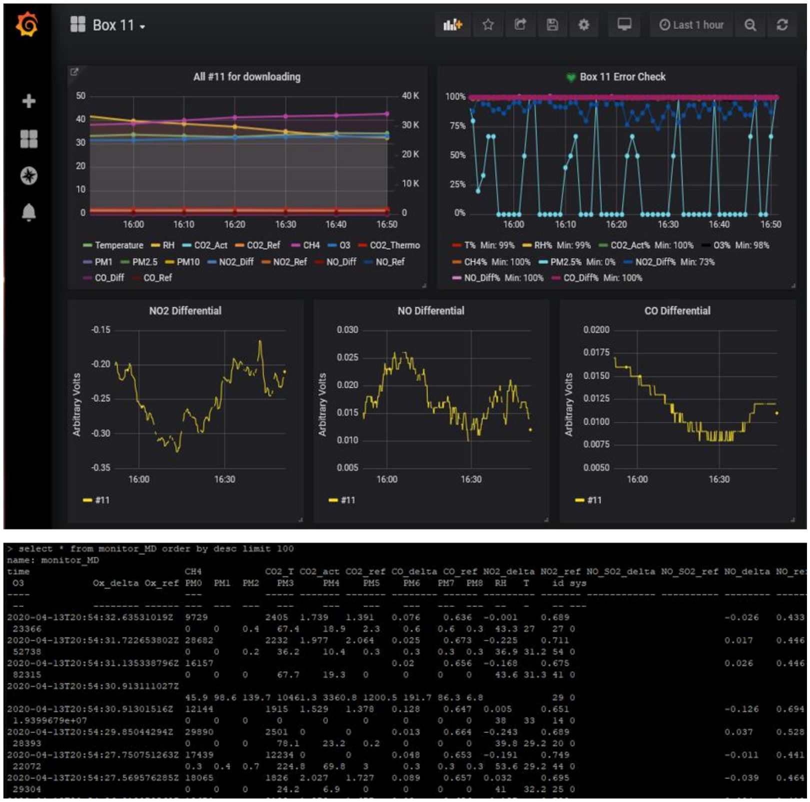

Figure S5: Data management and visualization software to see real time measurements. Grafana (**a**) is an open source data visualization platform allowing us to interface directly with our data management software InfluxDB (**b**) which is accessed through the secure shell PuTTY.

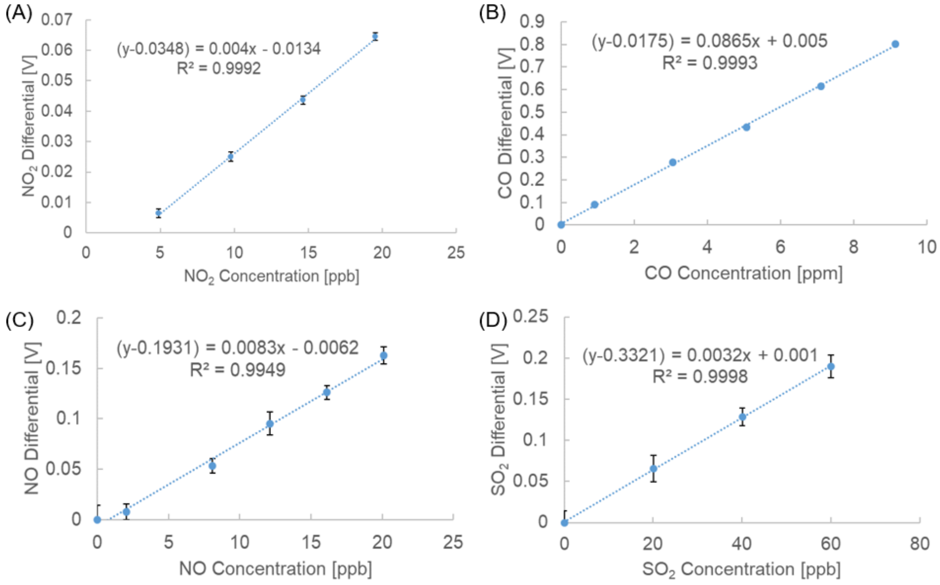

Figure S6: Demonstration of linearity of electrochemical sensor calibration. Error bars represent standard deviation of signal over several minutes of exposure to the target gas at a given concentration. Note that this is for the calibration of one sensor and there is variation in slopes among sensors.

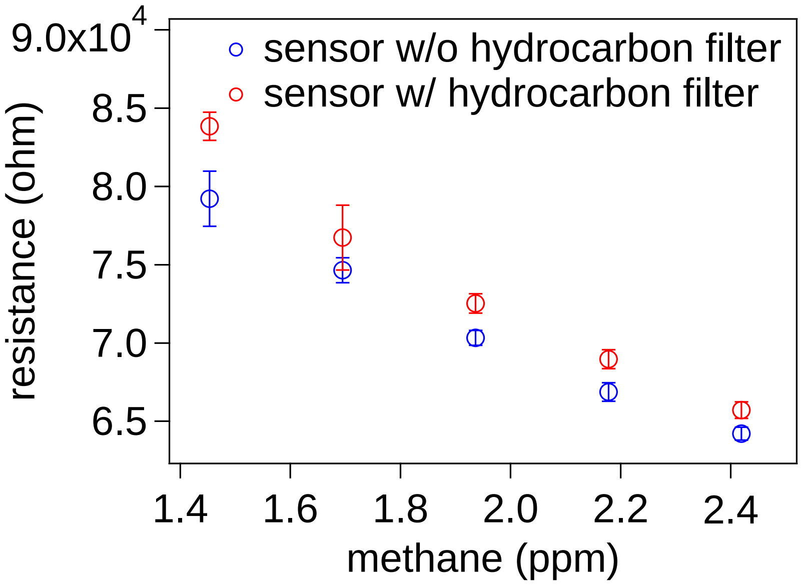

Figure S7: Calibration data for the Figaro TGS 2600 CH_4 sensor. A linear response is observed in typical ambient or urban background levels both with and without an activated carbon cloth filter to remove cross-sensitivity to VOCs.

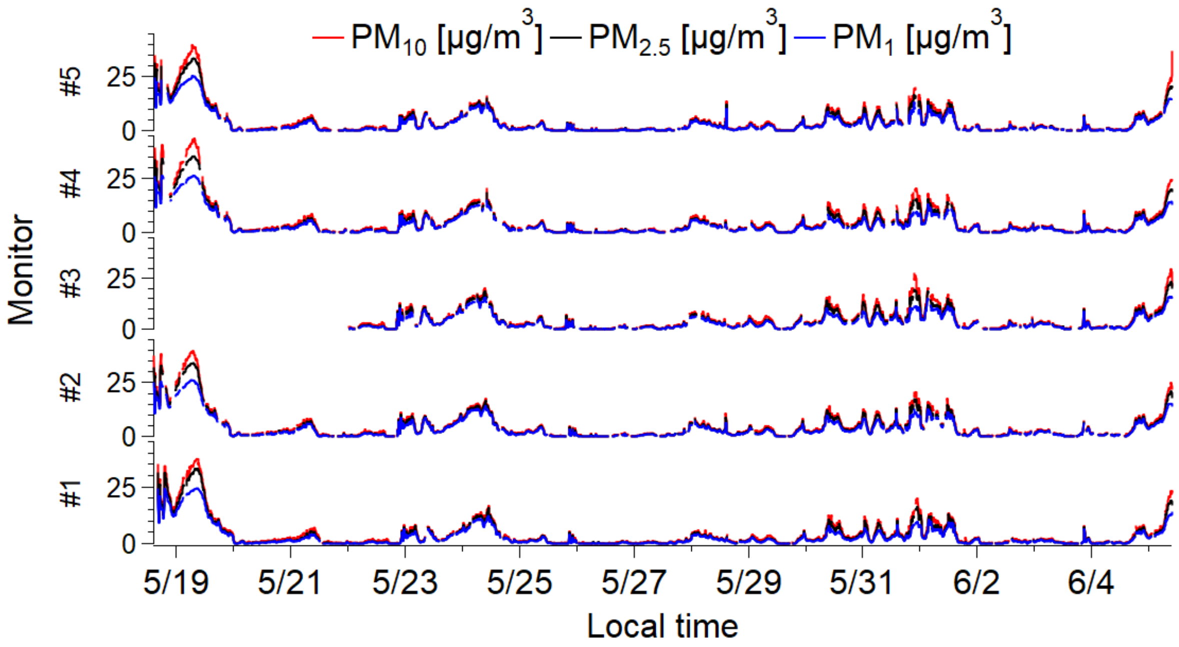

Figure S8: PM sensor intercomparison (raw data) for 5 co-located monitors over 2.5 weeks in New Haven, CT (accompanies data shown in Fig. 2b).

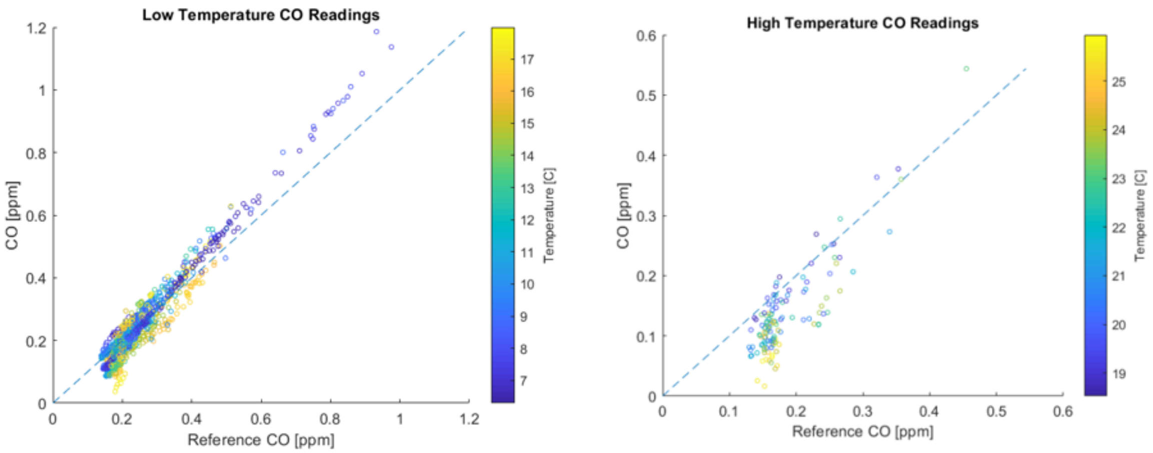

Figure S9: CO comparisons to reference instrument for low (<18 °C, left) and high (>18 °C, right) temperature readings. This accompanies Fig. 4 in the main text.

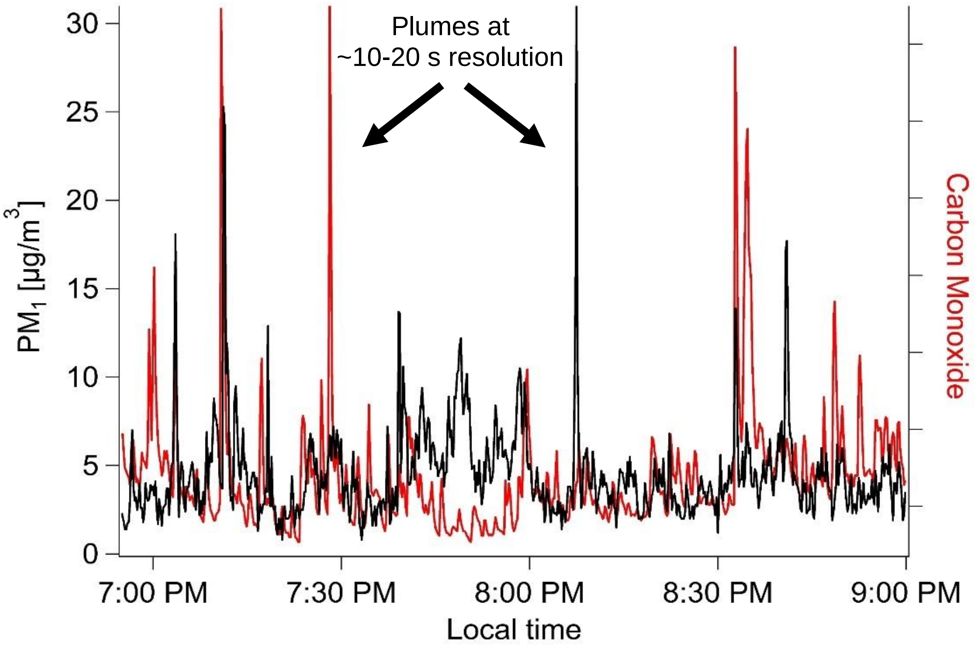

Figure S10: Additional examples of field data with fast monitor response times to concentration changes demonstrated roadside with motor vehicle spikes in PM_1 and raw CO differential signal at 10-s resolution.

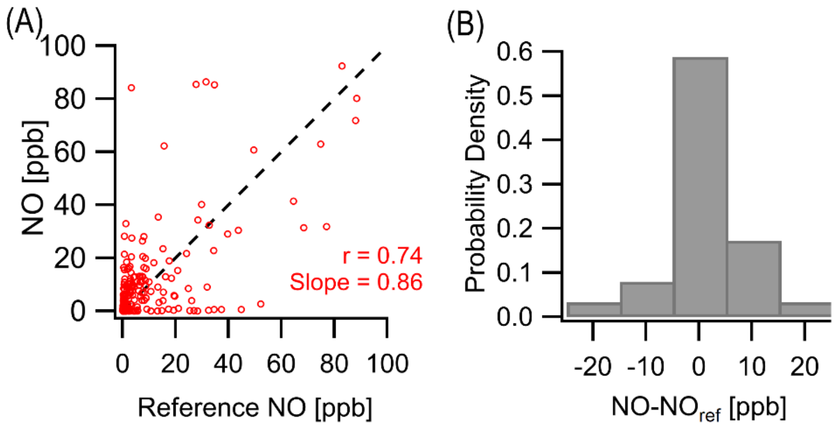

Figure S11: NO sensor regression (**a**) and probability density (**b**) from a two-week field deployment in New Haven, CT. The reference instrument comes from the near-road DEEP Criscuolo Park site (1.6 km away from sampling location in downtown New Haven). Given the generally low NO levels during the sampling period the probability density is shown as the MBE rather than the ratio of sensor to reference as in main text figures. Nearly 60% of data points fall within ±5 ppb of the reference at a 1 h resolution. Accompanies the data shown in Fig. 7.

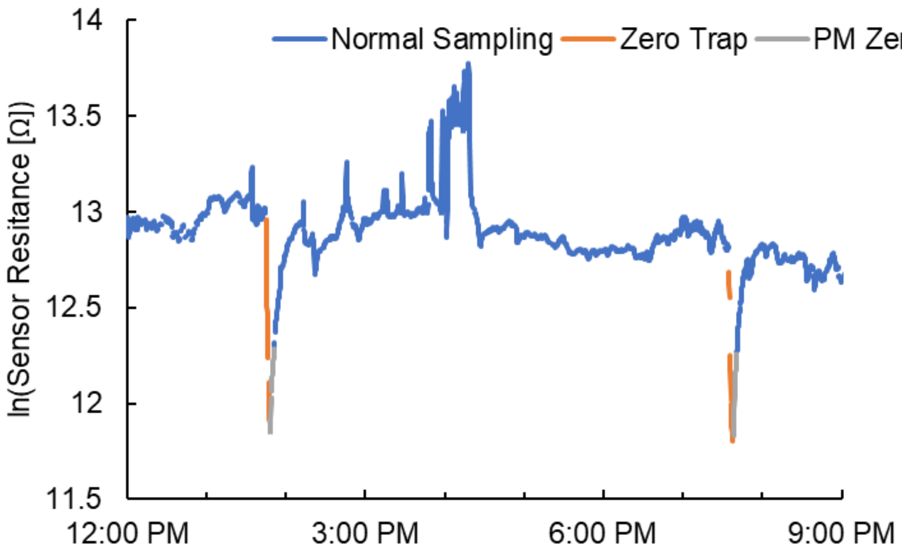

Figure S12: Performance of the zero trap for O_3 removal via steel wool and activated carbon shown as raw O_3 sensor resistances. Two tests during a mid-July afternoon in Baltimore, MD show a rapid decrease in sensor response through the zero trap (orange) relative to normal sampling and other calibration steps. Note: data are shown on a logarithmic scale due exponential response to concentration.

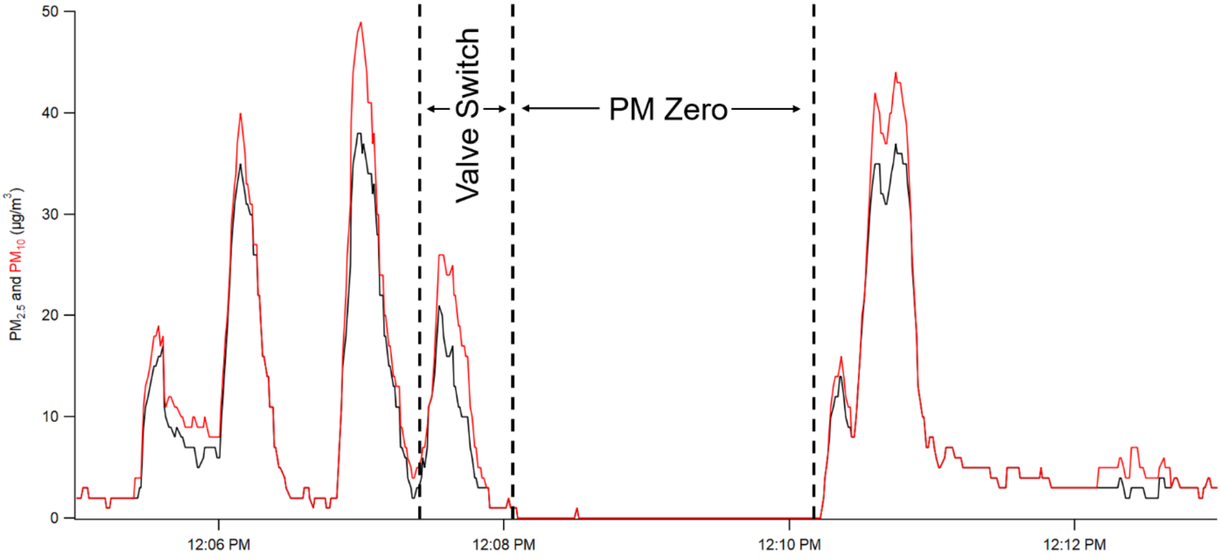

Figure S13: Raw $PM_{2.5}$ (black) and PM_{10} (red) sensor zero near food-cooking carts in New Haven, CT. After the valve switch, a short lag occurs (i.e. to purge inlet) before the sensor is zeroed for two minutes.

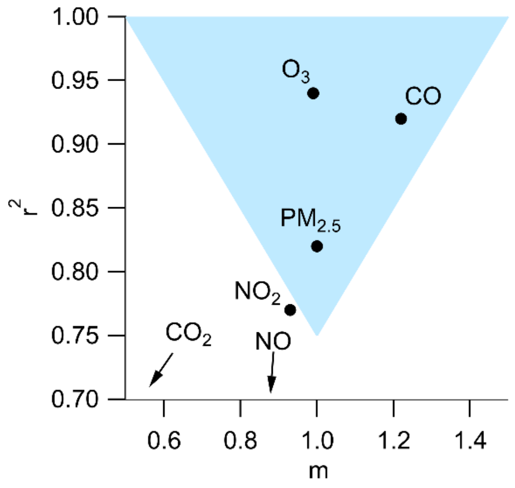

Figure S14: Sensor r^2 and slope values for collocation data. Blue shaded triangle represents the "best selection region" from Karagulian et al. (2019). Three of our measurements lie within this region (O_3, CO, and $PM_{2.5}$), with one along the edge (NO_2). Two pollutants (CO_2 and NO) did not fall into the region partially due to not having a true co-location comparison (reference sites were 2.7 and 1.6 km away from sampling location, respectively).
